# Characterization and purification of *Pseudomonas aeruginosa* phages for the treatment of canine infections

**DOI:** 10.1186/s12866-025-04005-4

**Published:** 2025-05-14

**Authors:** Anne Dalponte, Viviane Filor, Antina Lübke-Becker, Marcus Fulde, Thomas Alter, Mathias Müsken, Wolfgang Bäumer

**Affiliations:** 1 Institute of Pharmacology and Toxicology, School of Veterinary Medicine, Freie Universität Berlin, Berlin, Germany; 2 Institute of Microbiology and Epizootics, School of Veterinary Medicine, Freie Universität Berlin, Berlin, Germany; 3 Institute of Food Safety and Food Hygiene, School of Veterinary Medicine, Freie Universität Berlin, Berlin, Germany; 4 Central Facility for Microscopy (ZEIM), Helmholtz Centre for Infection Research, Braunschweig, Germany; 5 Veterinary Centre for Resistance Research (TZR), School of Veterinary Medicine, Freie Universität Berlin, Berlin, Germany

**Keywords:** Phage therapy, *Pseudomonas aeruginosa*

## Abstract

**Background:**

*Pseudomonas aeruginosa* is an opportunistic pathogen that causes infections in both human and veterinary medicine, presenting significant challenges in treatment because of biofilm production and its intrinsic resistance. This problem is exacerbated by the increase in acquired antimicrobial resistance. Bacteriophage (phage) therapy has emerged as a promising alternative for treating infection classically treated with antibiotics, offering a targeted approach to combat this infection. This study aimed to evaluate the therapeutic potential of 7 phages, focusing on their suitability for treating canine infections, as well as their purification and safety analysis for therapeutic use.

**Results:**

Two self-isolated phages and five provided phages were analysed. All tested phages reduced bacterial load in vitro; however, their efficacy varied across different concentrations. The host range analysis revealed a spectrum between 9.8 and 68.6% of canine clinical *P. aeruginosa* isolates. In our in vitro tests 3 out of 7 phages were able to significantly reduce the biofilm biomass, achieving reductions up to 93.38%. The sequence analysis did not discover known virulence factors and genes connected to antimicrobial resistance mechanisms. The self-isolated phages were classified as lysogenic, whereas the other phages had a lytic infection cycle. Through the purification of the phages, high-titre phage preparations (> 10^11^ PFU/ml) were generated with high stability for at least 1.5 years. The tested endotoxin units are below the regulatory limits.

**Conclusion:**

Investigating phages as alternative treatment option seems promising with lytic phages covering a broad host range and a genomic potential for biofilm degradation. These findings support the development of phage cocktails as a targeted alternative for treating canine *P. aeruginosa* infections, particularly in cases of antibiotic resistance, and highlight the importance of selecting well-characterized lytic phages for therapeutic efficacy and safety.

**Supplementary Information:**

The online version contains supplementary material available at 10.1186/s12866-025-04005-4.

## Background

*Pseudomonas aeruginosa (P. aeruginosa)* is an opportunistic pathogen that poses a major challenge in both human and veterinary medicine because of its high intrinsic resistance to commonly used antibiotics [1], ability to form biofilms [2, 3] and increased acquired antimicrobial resistance [4]. This phenomenon promotes the disease progression and contributes to the development of chronic infection [5–7].

The One Health concept emphasizes the interrelation of human, animal and environmental health, indicating that zoonotic pathogens and antimicrobial resistance can spread across species. The effective management of zoonotic pathogens such as *P. aeruginosa* is essential to prevent cross-species transmission and ensure public health. The pathogen can be transmitted bidirectionally through direct contact [8], through shared healthcare environments [9], environmental contamination and food chain transmission [10]. Although the transfer from livestock animals to humans is of particular interest in the One Health concept, numerous studies have demonstrated the infection of pet owners with (antimicrobial-resistant) pathogens from companion animals such as dogs, due to their close interaction within household [11–14]. This zoonotic potential is supported by a recent One Health-based investigation conducted in northern Portugal, analyzing 737 *P. aeruginosa*-strains from humans, domestic animals and aquatic environments. Genetic similarities and overlapping high risk clones across the sectors have been found, supporting the interconnectedness between the reservoirs [15].

Owing to its global threat, especially in health care settings, resistant *P. aeruginosa* is classified as a high-priority pathogen on the WHO Bacterial Priority List 2024 [16].

*P. aeruginosa* is a common cause of soft tissue infections such as wound infections, pyoderma, otitis, and urinary tract infections in companion animals [17]. The overall prevalence of *P. aeruginosa* in dogs and cats has been reported to be roughly 8% [18]. The most common canine infection is otitis with a prevalence ranging from 25 to 41%. The resistance spectrum in dogs and cats shows a wide variation, influenced by study design, unknown prior antibiotic use and susceptibility testing method applied. The European Food and Safety Authority (EFSA) evaluated the antimicrobial resistance and provided an overview of resistance levels in the European Union and the development of resistance in *Pseudomonas* for canine infections [19]. For gentamicin, the resistance ranged widely from 4 to 62% and for enrofloxacin from 4 to 68%, both antibiotics are commonly used in *P. aeruginosa* treatment in veterinary medicine. The major issue is the carbapenem resistance, carbapenems are key antimicrobials for human medicine. In several studies, high resistance prevalence has been reported, although this antimicrobial class is not used in veterinary medicine. Thus, it is concluded, that *P. aeruginosa* poses a public health risk due to its zoonotic character and increase in resistance development [19].

The emergence of antimicrobial resistance is accelerated through the excessive or improper use of antibiotics in human and veterinary medicine, rendering the development of alternative treatment options such as phage therapy urgently necessary. Phages are promising tools for eliminating multidrug-resistant and biofilm-producing bacteria [20, 21]. Biofilms form a diffusion barrier, decreasing the antimicrobial susceptibility of bacteria in a biofilm up to 1000-fold compared with that of planktonic cells due to the formation of multicellular structures [22, 23]. The combination of phages and antibiotics provides a promising strategy in treating infections. Phages with biofilm-degradation capabilities can destroy the extracellular matrix, and thereby increasing the permeability of the antibiotics to the inner layer and inhibiting the quorum sensing activity [24]. The possible synergistic effect of phages with some classes of antibiotics led also to a resensitization of the bacteria to antibiotics, providing an effective solution for the treatment of phages that are not treatable with antibiotics alone [25].

Phages have been used to treat humans and animals since the last century, but while they have been largely displaced in Western countries, phage therapy is still practiced in Eastern European countries (e.g., Georgia) [26]. Notably, it recently gained in interest also in the Western countries due to the global antibiotic resistance crisis. Biofilm formation and antimicrobial resistance are two major challenges in treating *P. aeruginosa* infections, leading to chronic infections.

To date, there are few publications on the clinical use of phages. Experimental work in mice for the treatment of systemic *P. aeruginosa* infection resulted in the survival of all the mice [27], and phages also showed clear effects against *P. aeruginosa* in a mouse model of sepsis [28]. Topically applied phages have shown good efficacy in models of infected wounds following burn injury [29].

For the moment, there are multiple phase I and phase II clinical trials with phages in the USA. For now, the FDA has not approved any phage preparation for clinical use. Apart from the clinical trial, also the use as compassionate therapy is possible. A retrospective study [30] evaluated the outcome of 100 patients treated with personalized phage therapy, reporting a clinical improvement in 77.1% of the infections and a bacterial eradication in 61.3% underscoring the potential of the phage therapy as a safe and effective therapeutic. The phages were co-administrated with antibiotics in most of the cases, and in vitro synergy tests were performed to identify the most effective combinations. Also in veterinary medicine, there is a lack of comparable blinded studies, but numerous case studies supported the efficacy of phages [31, 32].

The major challenge in phage therapy is finding efficient phages for therapeutic application, as they should have a wide host range, good propagation dynamics and a lytic infection cycle. The absence of virulence factors and antimicrobial resistance genes is essential to ensure safe application. The antibiofilm activity [33] of the phages would also be advantageous.

In our studies, we investigated several phages against *P. aeruginosa* with a particular focus on a broad host range, biofilm degradation ability, preparation stability and the lytic infection cycle. Lysogenic phages are not suitable for phage therapy, as they can integrate into the bacterial genome as prophages. Their integration allows them to be transmitted vertically through bacterial cell division. Additionally, lysogenic phages can encode antiphage systems that can affect the effectiveness of therapy [34]. Through characterization of the phages, the selection of the most suitable phages for the topical treatment of dogs can be determined.

## Method

### Phages and bacterial isolates

The phages were self-isolated or provided by Leibniz DSMZ. The phages provided by the DSMZ were JD05 (DSM27059), JG003 (DSM19870), JG004 (DSM19871), PTLAW1 (DSM105275) and PTLAW2 (DSM105276). These phages and the self-isolated phage AV001 were propagated using the *P. aeruginosa* strain IMT45060. The self-isolated phage AV002 was propagated using the strain *P. aeruginosa strain* DSMZ25641-1. All the bacterial isolates were provided by the Institute of Microbiology and Epizootics of the FU Berlin. The stain DSMZ25641-1 was provided by the DSMZ.

### Bacterial isolation and antimicrobial susceptibility testing (AST)

The *P. aeruginosa* isolates were recovered from canine samples submitted by veterinarians in Germany to the diagnostic laboratory of the Institute of Microbiology and Epizootics (Department of Veterinary Medicine, FU Berlin) between 2018 and 2022. Bacterial isolation and identification were performed according to standard microbiological procedures as described previously [35]. AST was performed according to CLSI standards [36, 37]. The breakpoint-specific MIC values of the antimicrobial compound amikacin, gentamicin, tobramycin, piperacillin/tazobactam, ceftazidime, marbofloxacin, enrofloxacin, imipenem, meropenem and ciprofloxacin, relevant for the treatment in human or veterinary medicine, were included in our analyses. Qualitative assessment of the MIC values was performed via the canine-specific breakpoints for amikacin, gentamicin, tobramycin, piperacillin/tazobactam, ceftazidime and cat-specific breakpoints for enrofloxacin (CLSI VET01S ED6:2023). For antimicrobial agents without a defined veterinarian-specific breakpoint, human breakpoints (CLSI M100 ED33:2023) were used. No CLSI breakpoints are available for the antimicrobial agent marbofloxacin. A total of 49 canine clinical *P. aeruginosa* strains were included in the examinations. Additionally, the phage propagation strains were tested.

### Phage isolation and transmission electron microscopy

Phages were isolated by further processing pooled samples of dog faeces and saliva samples, soil samples and environmental samples from a small animal clinic. Samples were presoaked in SM buffer (100 mM NaCl, 8 mM MgSO₄, 50 mM Tris-HCl [pH 7.5], 0.01% gelatine). The supernatant of the centrifuged (4 500×g, 40 min, 15 °C) samples was filtered through a 0.22 μm filter (CME) and mixed 1:5 with LB broth (×5) and ~ 1% volume of one of the 10 different *P. aeruginosa* isolates (OD_600_ 0.5) [see Additional file 1]. Each supernatant-isolate mixture was incubated separately for 24 h (37 °C, 200 rpm), followed by the centrifugation and filtration step described previously. The presence of phages in the filtrate was tested via the plaque assay described in [38]. Briefly, 100 µl of the supernatant was mixed with the bacterial culture and 0.3% top agar (LB media), poured onto a plate with 1.2% bottom agar (LB media plus 1 mmol Ca and 1 mmol Mg), and incubated overnight at 37 °C. Phages were purified with a minimum of three cycles of single-plaque isolation followed by a plaque assay of the serial dilutions. To visualize the phages under a transmission electron microscope, we prepared negative-stained samples. Therefore, phage particles were allowed to attach to a grid with a carbon layer, washed, stained with 2% uranyl acetate and heat-dried. The samples were examined with a Libra 120 transmission electron microscope (Zeiss, Oberkochen, Germany) at calibrated magnifications.

### Genome sequencing, genome assembly and annotation

For whole-genome sequencing (WGS), the phage genome was extracted with the Norgen phage DNA isolation kit, followed by library preparation with the Nextera XT DNA Library Preparation kit. Two × 300 bp paired-end sequencing of 200-fold multiplexes was performed on the Illumina MiSeq platform. For sequence assembly, the Illumina reads were trimmed with *Trim Galore* (v0.6.6) [39] and quality checked with FastQC [40]. De novo assembly was carried out via SPAdes (3.15.0) [41]. The sequencing data of the phages JG003, JD05, PTLAW1 and JG004 were provided by the DSMZ. The annotation of the genomes of all the phages was performed on the Galaxy CPT platform public server (https://cpt.tamu.edu/galaxy-pub) [42] via the structural workflow (v2021.02) and functional workflow (v2023.01). The infection cycle of the phages was additionally confirmed through PhageAI S.E. (https://phage.ai/). To identify putative toxins, antibiotic resistance alleles and virulence factors, the sequences were compared through ABRicate (https://github.com/tseemann/abricate) [43] with the up-to-date databases Comprehensive Antibiotic Resistance Database (CARD) [44], ResFinder [45], AMRfinder [46], Bacterial Antimicrobial Resistance Reference Gene Database [47], Antibiotic Resistance Gene-ANNOTation (ARG-ANNOT) [48], MEGARes 2.0 [49]., PlasmidFinder [50], and virulence factor database (VFDB) [51]. The whole genome sequence with annotation is deposited at GenBank (National Center for Biotechnology Information) using the following accession numbers: AV001: PQ349086; AV002: PQ349087; JD05: PQ676539; PTLAW1: PQ879542; PTLAW2: PQ798946; JG003: PV105456; JG004: GU988610 [52].

### Phylogenetic analysis

To identify the closest relatives at the phylogenetic level, the entire genome sequence of each phage was used as an input for the Viptree server (version 4.0) (https://www.genome.jp/viptree, accessed October 2024) [53], which classifies the virus on the basis of genomic-wide similarities computed by tBLASTx [53]. The 40 most similar phage genomes were used as inputs for the VICTOR web service (https://victor.dsmz.de, accessed on November 2024) to generate a phylogenetic tree, a method for the genome-based phylogeny and classification of prokaryotic viruses [54]. For the calculation of the genetic distance for the phylogenetic tree generation the D0 formula was used, as recommended for the analysis of nucleotide sequences by VICTOR. In accordance with the ICTV recommendations, the phage taxonomy was further delineated via VIRDIC (https://rhea.icbm.uni-oldenburg.de/VIRIDIC/, accessed on November 2024) [55], which aligns the 20 most similar phages. The phages were classified into the same species if they shared an average nucleotide identity (ANI) greater than 95% and 70% for the same genus. The genomes belonging to the same genus were aligned via Viptree.

### Phage host spectrum and efficiency of plating (EOP)

To determine the host range of the phages, a spot assay of the phages was performed on the 51 isolates as previously described. The host range was assessed using the same clinical canine isolates of antimicrobial resistance testing. The undiluted phage suspension was spotted in duplicate onto a two-layered agar plate composed of 1.2% bottom agar (LB medium with 1 mmol Ca and 1 mmol Mg) and 0.3% top agar (LB medium) mixed with the respective bacterial isolate [38]. After overnight incubation at 37 °C, the lysis zones were evaluated. For the determination of EOP, the phages that showed a lysis zone on the respective strain were retested in a spot assay by spotting 10-fold dilutions. The EOP was defined as the ratio between the calculated PFU/ml of the susceptible strain and the reference strain.

### Time-killing-assay

The lytic effect of the phages was tested by the time killing curve over 24 h. 100 µl of the phage propagation strain (1 × 10^8^ CFU/ml) were mixed with 50 µl of the phage-suspension at an MOI of 1, 0.1, 0.01 and 0.001 in a 96 well plate (F-bottom) and sealed with the Breath-Easy sealing membrane (Sigma). The microplate was incubated at 37 °C for 24 h with shaking for 30s every 5 min in a plate reader (Varioscan ALF Multimode Microplate Reader, ThermoFisher). The OD_600_ was measured every 10 min. The phages were tested alone and in combination with the phages of different genus. For data analysis the LB-control was subtracted. All experiments were performed in triplicate.

### Biofilm degradation assay

The host bacteria were grown to an OD_600_ of 0.1, transferred to a 96-well plate (U-bottom) and cultivated at 37 °C for 20 h. The wells were washed three times with sterile PBS to remove planktonic bacterial cells. Different phage concentrations (10^8^, 10^7^, 10^6^, and 10^5^ PFU) were applied to the wells, which were subsequently incubated for an additional 24 h. The wells were washed three times with *aqua bidest*. To quantify the total biomass of the biofilm, the wells were stained with 0.1% crystal violet solution and incubated for 15 min. After the wells were rinsed three times (with *aqua bidest*), the crystal violet was dissolved in 30% acetic acid on an orbital shaker. Adsorption was measured at 550 nm (flat bottom). All the experiments were carried out in triplicate.

### Physical phage stability

To test phage stability, 250 µl of phage suspension with a defined titre (10^8^ PFU/ml) was incubated at different temperatures (4 °C, 20 °C, 32 °C, 39 °C, 50 °C) and pH values (5, 7.4, 9). Samples were taken after 30 min and after 6, 16, and 24 h. The tenfold dilutions of each phage under each condition were tested in duplicate via a spot assay as previously described. The titre was converted into % values using the initial phage titre as 100%. Temperatures were chosen according to the storage temperature (4 °C), room temperature (20 °C), canine skin surface temperature (32 °C [56]) and canine auricular temperature (39 °C [56, 57]). The temperature of 50 °C was included to represent the condition of the top agar when adding the phages during plaque assays. The pH-values were chosen to reflect a spectrum of gel formulations with varying pH values.

### Phage amplification and purification

LB broth was inoculated with the precultured host bacterial strains and grown at 37 °C with shaking to an OD_600_ of 0.2, with a final MOI of 0.1, and cultivated overnight. The obtained lysate was centrifuged twice (8 000 ×g, 4 °C, 45 min). The supernatant was filtered through 0.45 μm and 0.22 μm membranes. The diafiltration of the phage lysates was performed with a CFF system (Vivaflow 50R, Hydrosat Membrane, MWCO 100 000, Sartorius). A 10-fold concentration was achieved by recirculating the phage lysate though the system. Diafiltration was performed with PBS or SM-Buffer (supplemented with Ca and Mg) until the phage lysate became clear and colourless. For density gradient centrifugation, the phage suspension was loaded on a CsCl step gradient (densities of 1.4, 1.5 and 1.6) and centrifuged at 40 000 ×g for 16 h at 4 °C (SW41 rotor in Optima XPN-80 (Beckman)). The band containing the phages was collected and stored at 4 °C. The samples were placed on a dialysis membrane (Spectra/Por MWCO 100 000) and dialyzed with three buffer changes against 300x the volume of aqua bidest and PBS or SM buffer for a total of 24 h. The phage preparations were stored at 4 °C in dehydrogenated glass bottles (Lonza). The endotoxin concentration was tested with diluted samples in duplicate via the chromogenic Endotoxin Quant Kit (Pierce) according to the manufacturer’s protocol, which uses a high standard (1–0.1 EU/ml). The endotoxin unit and the amount of the produced doses were calculated for a dose of 10^8^ PFUs. Phage stability was tested after 1.5 years by spot assay.

### SDS‒PAGE of the purification steps

To verify the removal of the bacterial protein components via the purification steps, the denatured protein components were resolved via 1D SDS‒PAGE. The bacterial host culture samples and the sterile-filtered, diafiltered and dialyzed phage samples were diluted with Laemmli protein sample buffer (Roth) and denatured for 15 min at 95 °C. Proteins were resolved in a 12% polyacrylamide gel. The gel was loaded with 5 µl of the protein ladder (PageRuler™ Plus Prestained Protein Ladder, 10 to 250 kDa) and 15 µl of the sample and electrophoresed at 80 V with the Protean System (Bio-Rad). Proteins were visualized with PageBlue™ Protein Staining Solution (Thermo Scientific), and gel images were acquired via the Imager CHEMI Premium (VWR).

### Statistics and data analysis

The results of bacterial cell lysis are presented as the mean ± standard deviation (SD). The percent reduction in biofilm biomass was calculated by normalizing the optical density at 550 nm of the test samples relative to the control without phages. For the percent biofilm biomass reduction and endpoint (24 h) comparison of the growth kinetics, One-Way ANOVA with Dunnett’s multiple comparisons test was performed. The significance of an error probability of at least 5% was considered significant. The diagrams of the biofilm reduction are presented as floating bars with minimal and maximal values with the line at the mean. The statistical evaluation and generation of the graphs were performed with GraphPad Prism (version 10.0.2, GraphPad Software, Inc.). Figure 10 was partially created with biorender.com.

## Results

### Bacterial isolation and antimicrobial susceptibility testing (AST)

**Fig. 1:**
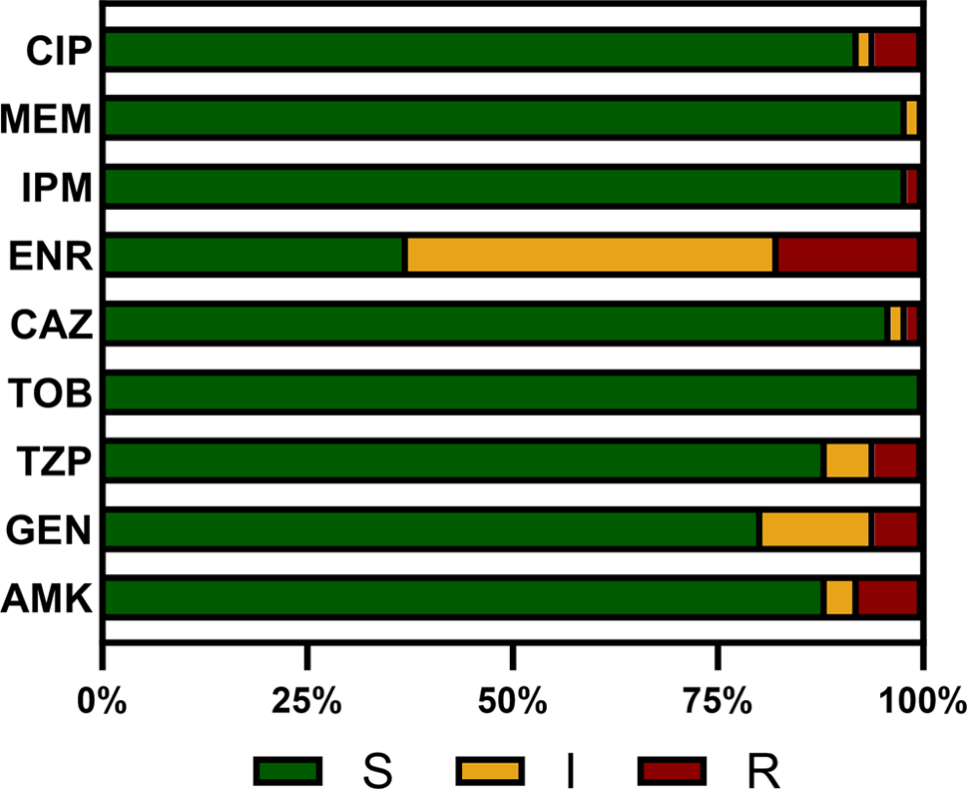
Antimicrobial susceptibility of the *P. aeruginosa* isolates. AMK, amikacin; GEN, gentamicin; TZP, piperacillin-tazobactam; TOB, tobramycin; CAZ, ceftazidime; ENR, enrofloxacin; IMP, imipenem; MEM, meropenem; CIP, ciprofloxacin; S, susceptible; I, intermediate; R, resistant. Marbofloxacin not shown, due to the missing breakpoints. *n* = 51. Susceptibilities were assigned according to CLSI guidelines

The *P. aeruginosa* isolates were recovered from canine species (*n* = 51) from different sources (Table 1) and subsequently tested for their antimicrobial susceptibility according to the CLSI standard [58]. The resistance to enrofloxacin was the highest at 17.65% (*n* = 9/51), whereas the resistance to the other antibiotics was comparatively low: amikacin (7.84%, *n* = 4/51), gentamicin (5.88%, *n* = 3/51), piperacillin/tazobactam (5.88%, *n* = 3/51), ceftazidime (3.92%, *n* = 2/51) and ciprofloxacin (5.88%, *n* = 3/51). No resistance to tobramycin or meropenem was detected. One of the tested strains was resistant to six of the tested agents, including imipenem (> 16 µg/ml). The test for the expression of carbapenemase in this strain was negative [see Additional file 2]. Owing to the unavailability of breakpoints for marbofloxacin, a qualitative interpretation is not feasible. For marbofloxacin, 15.69% of the strains presented an MIC value ≥ 4 µg/ml. The resistance level of the strains is shown in Fig. 1. All breakpoint-specific MIC values are provided [see Additional file 3].

**Table 1:** Origin of the isolates for susceptibility testing

Origin	number
**Skin and soft tissue (SST)**	
–Ear swab	28
–Skin swab	3
–Conjunctival swab	4
–Nasal swab	1
**Total**	**36**
**wound and abscesses (wds/abcs)**	
**Total**	**10**
**Urogenital tract (UGT)**	
–Vaginal swab	1
–Urine sample	2
**Total**	**3**

Isolate classifications according to the CLSI

### Phage isolation and morphological analysis

**Fig. 2:**
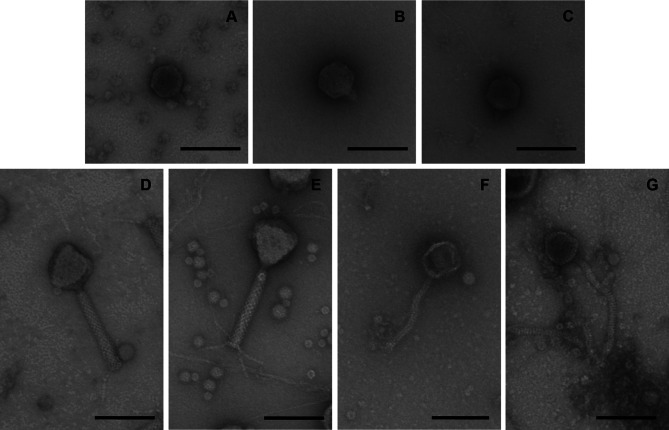
Transmission electron microscopy of the phages (**A**) JD05; (**B**) JG003; (**C**) JG004; (**D**) PTLAW1; (**E**) PTLAW2; (**F**) AV001; (**G**) AV002. The samples were negatively stained and magnified at 35k. The scale bar in each image represents 100 nm. According to their morphology the phages can be classified to the following morphotype: phages **A**-**C** podovirus like morphotype; **D**-**E** myovirus like morphotype, **F**-**G** siphovirus like morphotyp

The isolation of the two phages was performed via the purification of pooled samples from dog faeces, saliva samples, soil samples and environmental samples, followed by incubation with each of the 10 *P. aeruginosa* strains [see Additional file 1] separately. The presence of phages was confirmed via a plaque assay. The two self-isolated phages and the phages JD05, JG003, JG004, PTLAW1 and PTLAW2 provided by the DSMZ were included in further analyses. Analysis via transmission electron microscopy revealed that the isolated phages belong to the siphovirus like morphotype, which is characterized by a long, noncontractile tail. In contrast, the phages provided by the DSMZ belong to the podovirus like morphotype, with a short tail, and the myovirus like morphotype, with a contractile tail. The capsid diameters and tail lengths are listed in Table 2, and electron microscopy images are shown in Fig. 2.

**Table 2:** Morphology of the phages and sequencing findings

Electron microscopy				Sequencing			
Phage	Capsid diameter [nm]	Tail lenght [nm]	Morphotype	Genome size [kbp]	GC content [%]	Hypothetical proteins [%]	CDS
JD05	55.71 ± 2.3	17.33 ± 3.8	Podovirus like	53.39	51.97	64.63	82
JG003	61.13 ± 2.4	16 ± 1.9	Podovirus like	92.19	49.47	79.32	179
JG004	58.56 ± 2.57	16.38 ± 2.74	Podovirus like	93.02	49.25	76.30	173
PTLAW1	78.33 ± 2.77	150.33 ± 3.2	Myovirus like	66.11	55.60	86.17	94
PTLAW2	81.38 ± 4.0	148.25 ± 3.30	Myovirus like	66.24	55.61	61.62	99
AV001	60.55 ± 3.96	150.5 ± 3.50	Siphovirus like	39.54	61.67	61.29	62
AV002	58.75 ± 3.61	206.27 ± 6.90	Siphovirus like	40.24	57.35	42.37	59

### Sequencing

The seven analysed phages had a wide range in genome size, from 39.54 kbp to 93.02 kbp. The CG content of the genomes ranged from 49.25 to 62.2%, indicating that the smaller genomes tended to have a greater CG content than did the larger ones. The nucleotide sequences of the phages were compared with those in common databases of virulence factors and antimicrobial resistance factors, and the functions of the proteins were annotated to identify lysogenic genes. No known virulence-associated proteins, toxin proteins or antimicrobial resistance factors have been identified, revealing that phages are potentially safe for phage therapy. The phage JG004 was previously sequenced and analysed in another study [52]. Significant matches to genes linked to the lysogenic phages were found in the phages AV0001 (gene 7; 55; 59) and AV002 (gene 1; 13; 19; 20), suggesting that these phages have a lysogenic cycle, whereas in the other phages, no proteins linked to the lysogenic cycle were found. These lytic and lysogenic classifications were also confirmed by PhageAI. The genomes of the phages were screened for genes that are linked to the ability to degrade biofilms according to the results of the biofilm degradation assay. The phage JD05 encodes the genes lysozyme, endonucleases, exonucleases and glutamine-fructose-6-phosphate aminotransferase. The phage JG003 encodes the genes encoding endolysin, a putative exodeoxyribonuclease, a phosphoesterase, a putative cell wall hydrolase and an ATP-dependent Clp protease proteolytic subunit. The phage PTLAW1 encodes the genes putative chitinase, lytic transglycosylase and exonuclease. The annotation tables of the phages are provided in Additional file 4.

### Phylogenetic analysis and classification

**Fig. 3:**
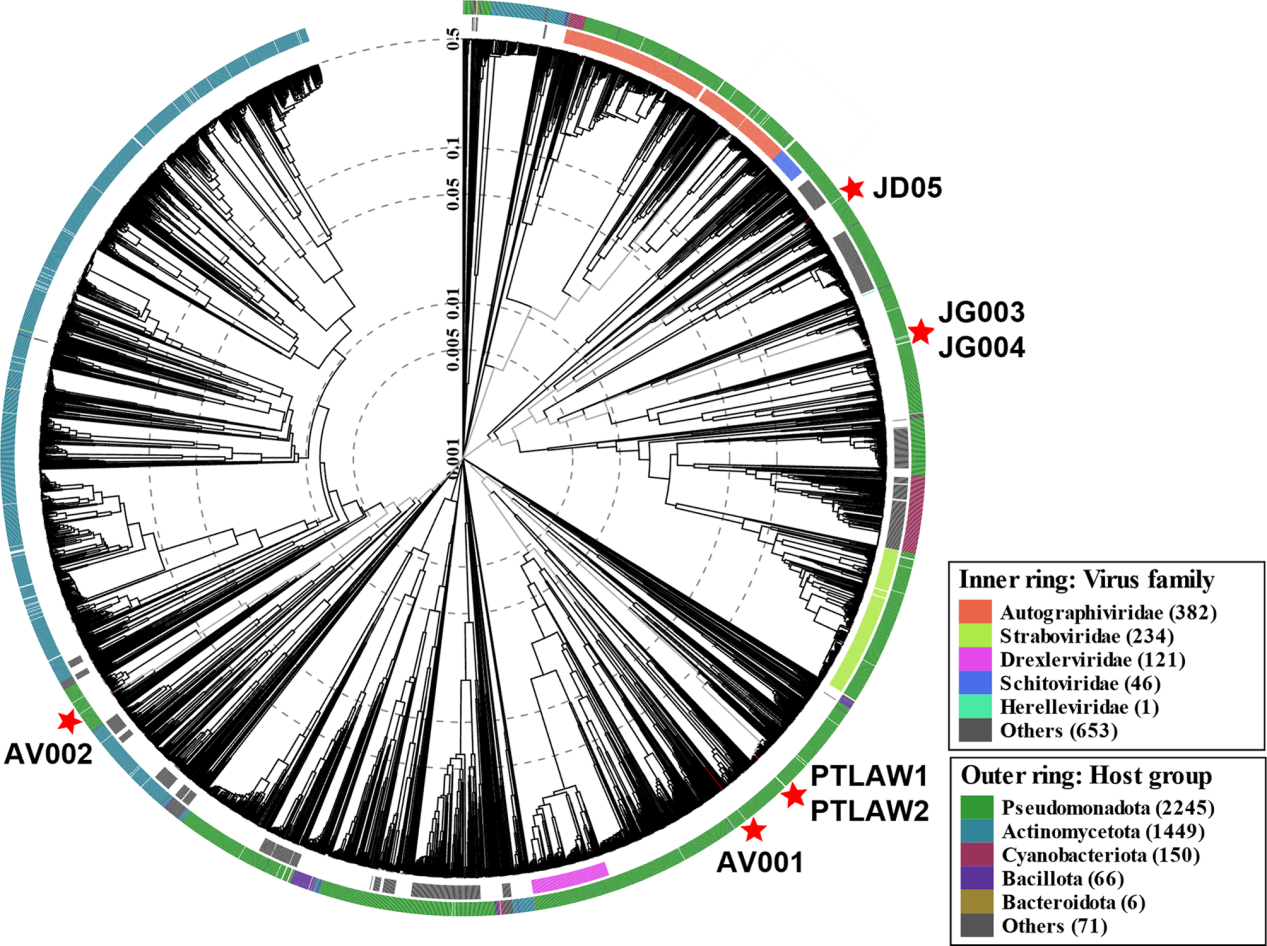
Viral proteomic tree. Phylogenetic analysis of the phages. The tree was generated by the VIPTree server. The inner ring represents the virus family, the outer ring the host group. The branch length was logarithmically scaled from the root of the proteomic tree. The tree is generated based on genome-wide similarities as determined by tBLASTx. The analysed phages are highlighted by red stars

Through VipTree (version 4.0), the results of the whole-genome sequencing were compared with tBLASTx with the database and ranked according to their genomic similarity (SG), and their relationships were visualized in a circular proteomic tree (Fig. 3).

**Fig. 4:**
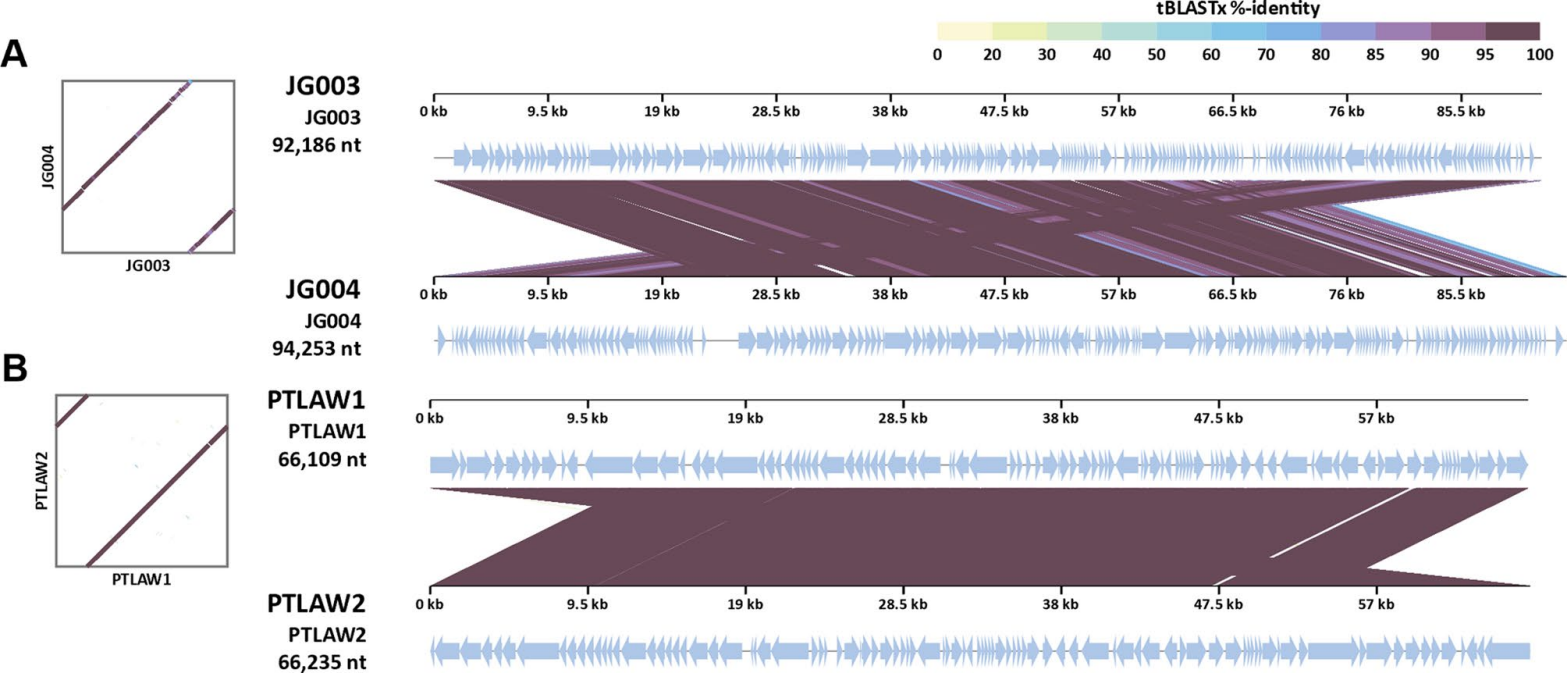
Vip-tree alignment of the phages JG003 with JG004 and PTLAW1 with PTLAW2 belonging to the genus Pakpunavirus and Pbunavirus, respectively. The colour of the bands between the seven genetic maps shows the percentage of identity between each sequence as indicated in the legend at the bottom of the figure

The phages were classified into their taxonomic group through VICTOR and VIRIDIC, on the basis of their intergenomic distance. The isolated phage AV002 likely belongs to the genus Beetrevirus, whereas for the phage AV001, no clear genus classification was possible. The phages JG003 and JG004 were assigned to a cluster with Pseudomonas phages belonging to the genus Pakpunavirus. The phages PTLAW1 and PTLAW2 were clustered in the Pbunavirus group. The phage JD05 was clustered in a cluster of well-characterized Pseudomonas phages belonging to the genus Bruynoghevirus. The alignment of the phages belonging to the same genus revealed high genomic identity at the genomic level (Fig. 4). Detailed phylogenetic analysis of all the phages is shown in a separate document [see Additional file 5].

### Time-killing curve and biofilm degradation analysis

**Fig. 5:**
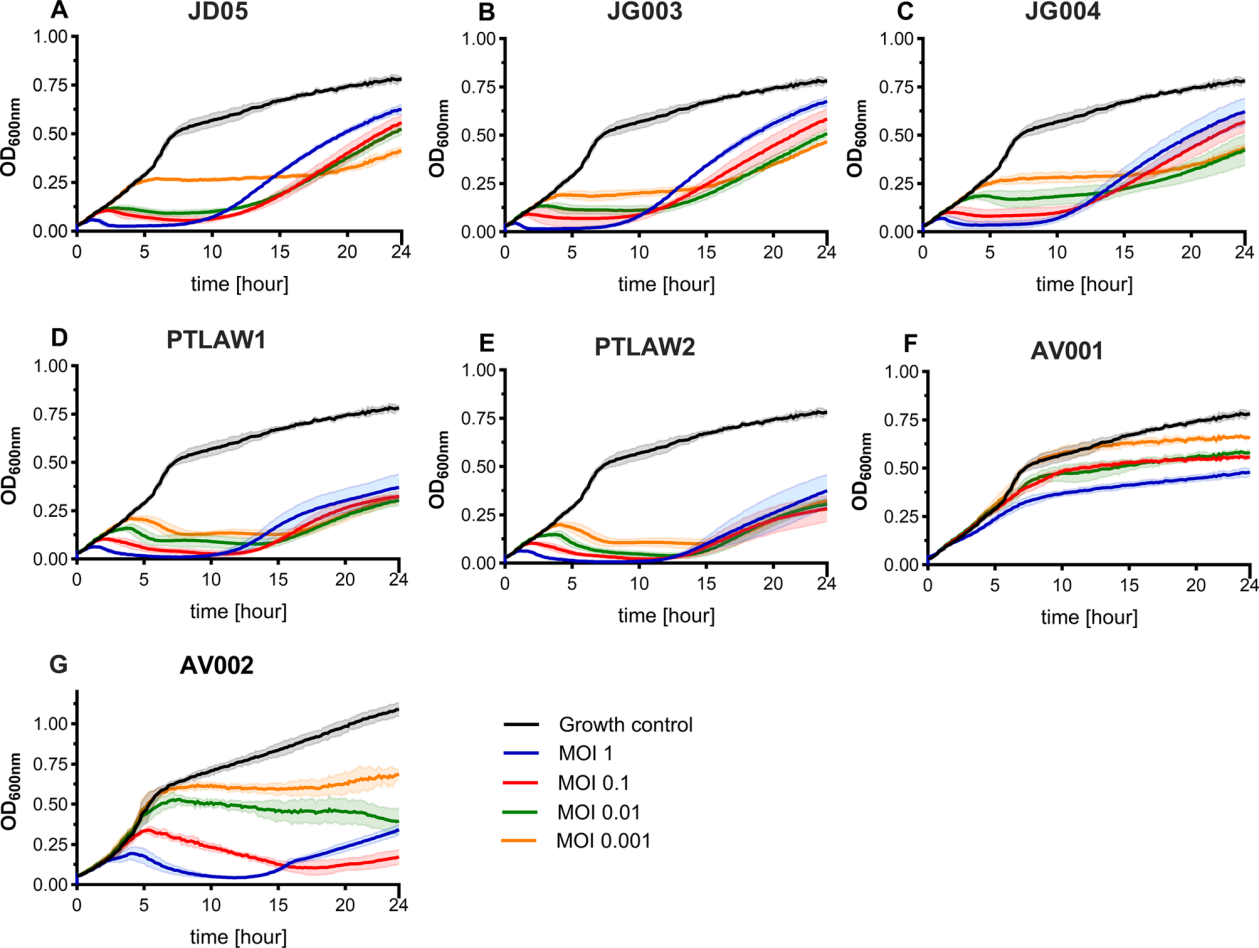
The kinetics of the time-killing curve. The lytic activity of the phages was tested on their propagation strain with different concentrations, stated as multiplicity of infection. (**A**) JD05; (**B**) JG003; (**C**) JG004; (**D**) PTLAW1; (**E**) PTLAW2; (**F**); AV001; (**G**) AV002. Error bars denote the standard deviation of at least three independent experiments

The lysis effect of the phages was evaluated alone and in combinations of phages with different genera by monitoring the OD_600_ over 24 h. The kinetics of the time-killing curve analysis are shown in Fig. 5. The bacterial strain without inoculation of phages (growth control) exhibited a continuous increase in optical density. In all experiments, the first inhibition of bacterial growth was dose-dependent and lasted for approximately 10 h. After this time point, bacterial regrowth observed in all the tested phages. For the lytic phages, the highest MOI resulted in the most inhibition at the beginning of the assay, but regrowth occurred earlier and was more pronounced than at the lower MOIs.

**Fig. 6:**
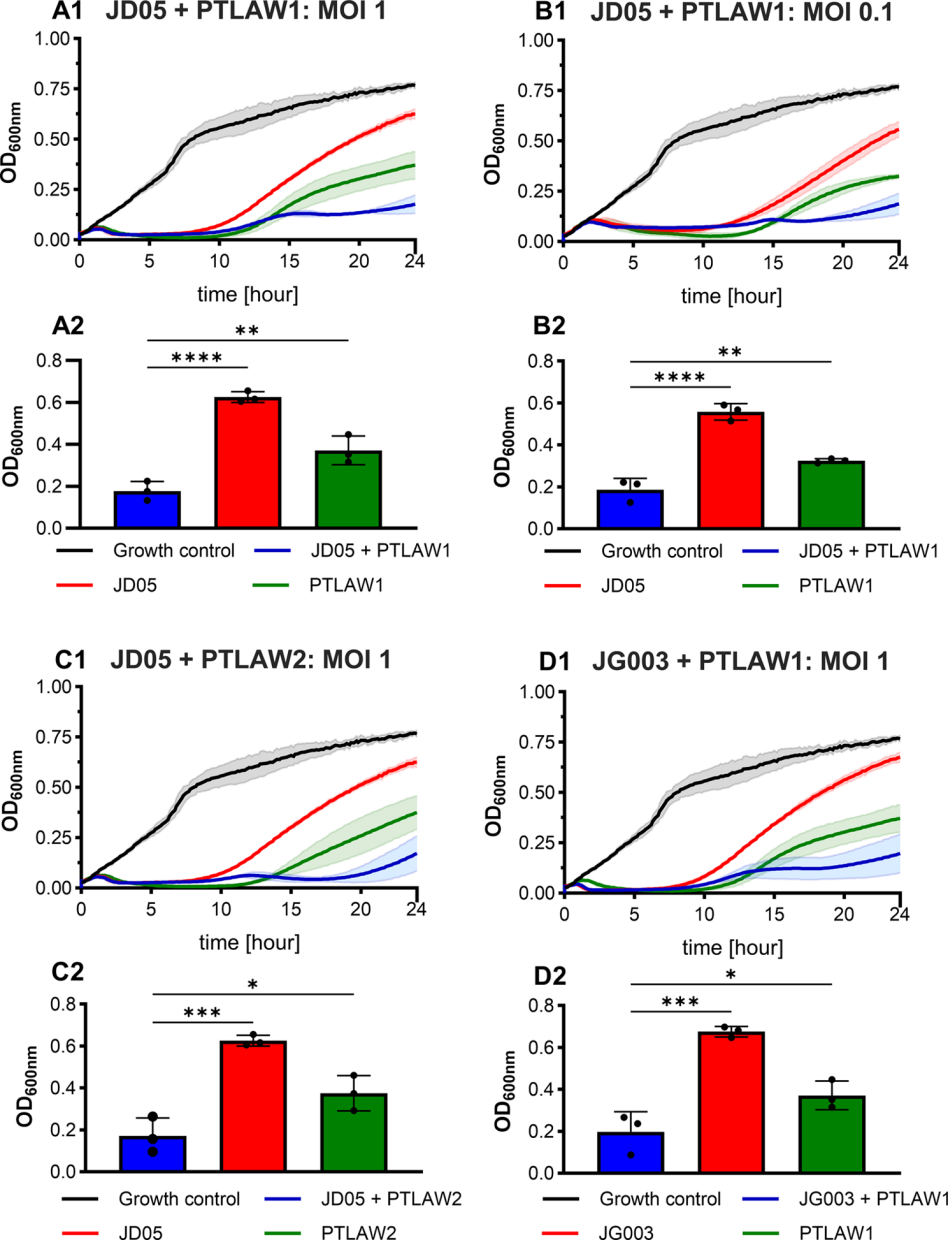
Effect on the time-killing effect by combination of two phages of different genus. Data are shown for combinations and MOIs that resulted in a significantly lower endpoint absorbance compared to each phage applied individually. (1) Growth kinetics over 24 h. (2) Endpoint absorbance measurement at 24 h. (**A**) JD05 + PTLAW1 MOI 1; (**B**) JD05 + PTLAW1 MOI 0.1; (**C**) JD05 + PTLAW2 MOI 1; (**D**) JG003 + PTLAW1 MOI 1. Growth curves and bar graphs represents the mean ± standard deviation. One-Way ANOVA with Dunnett’s multiple comparisons test was performed. * *P* ≤ 0.05; ** *P* ≤ 0.01; *** *P* ≤ 0.0005; **** *P* ≤ 0.0001

By combination of the phages, a dose-dependent reduction was similarly observed. However, except for two combinations (JD05 + JG003 and JD05 + JG004), a clear strong regrowth effect after approximately 10 h was not observed. Instead, the regrowth appeared more gradual. Nevertheless, regrowth of the bacteria was seen in all the combinations. Notably, for the same combinations, regrowth at the highest MOI was less pronounced compared to the single phage treatment. Comparing the endpoint absorbance between the combinations and the single phages (Fig. 6), significant lower absorbance comparing the single phages and the combinations were found: JD05 + PTLAW1 (MOI 1; MOI 0.1); JD05 + PTLAW2 (MOI 1); JG003 + PTLAW1 (MOI 1). The combination of three phages did not alter the growth kinetics compared to the corresponding two-phage combinations. The growth kinetics of all the phage combinations are shown in Additional file 6.

**Fig. 7:**
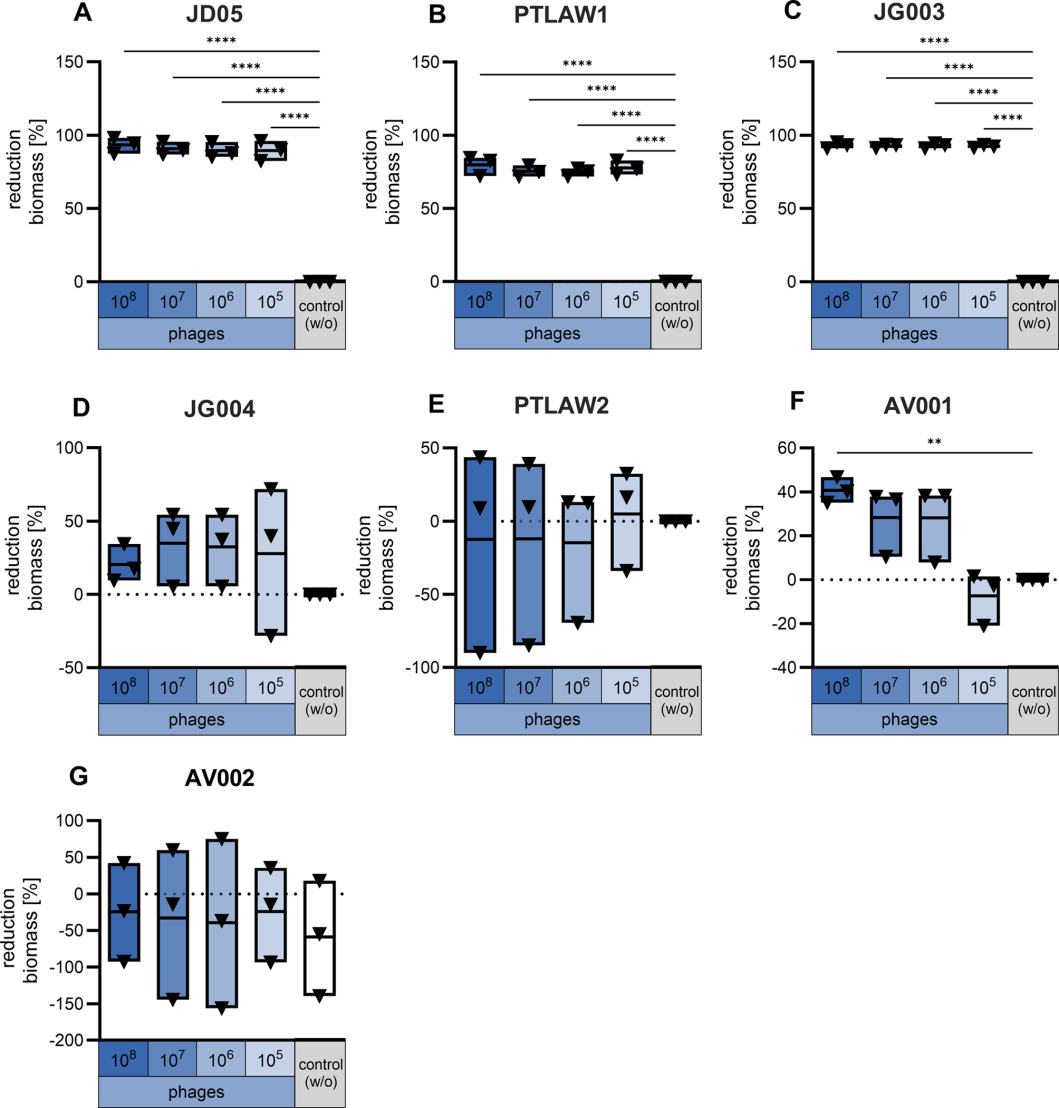
Biofilm degradation assay: Percent reduction of biofilm biomass compared to the untreated control (no phages). The total biomass of biofilm was stained with crystal violet and the optical density (OD) at 550 nm was measured and normalized for the control. (**A**) JD05; (**B**) PTLAW1; (**C**) JG003; (**D**) JG004; (**E**) PTLAW2; (**F**) AV001; (**G**) AV002. The phages **A**-**C** showed a significant reduction of biofilm biomass. One-Way ANOVA with Dunnett’s multiple comparisons test. ** *P* ≤ 0.01, **** *P* ≤ 0.0001. Data showed as floating bars with the mean

The ability of phages to degrade biofilms was tested via incubation of phages (10^8^, 10^7^, 10^6^, 10^5^ PFU) in a preformed biofilm, which was visualized via crystal violet staining of the biomass (Fig. 7). Compared with the control without phages. Calculated as percent reduction of the biomass compared to the control without phages. Three out of the 7 phages reduced the biofilm biomass significantly at all MOI. The phages JD05 and JG003 showed the highest reduction of 93.38% and 93.20%, respectively, while the phage PTLAW1 achieved 79.83% reduction.

### Host range and efficiency of plating (EOP)

**Fig. 8:**
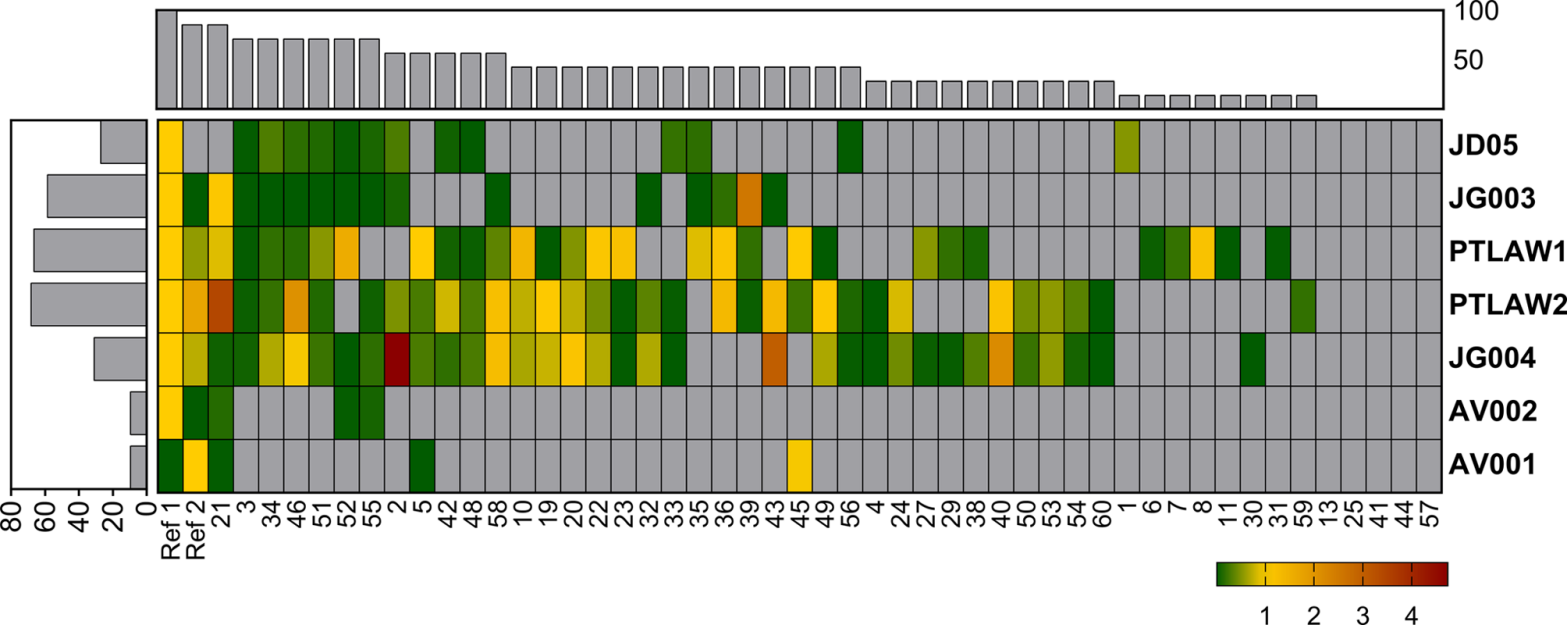
Host range of 51 clinical *P. aeruginosa* isolates, showed as heat map with color-coded efficiency of plating. The host range evaluation was performed by spotting serial dilutions of phages on the isolate. In the heatmap, the X-axis corresponds to the strains and the Y-axis to the phages. The color of the heatmap represents the EOP. The bar charts on the X- and Y-axes represent the number of infections for each strain and phage respectively

To determine host range and plating efficiency, undiluted phage suspensions were first spotted onto double layer agar, as previously described [38]. The strain-phage combination with the lysis zone was retested in 10-fold dilutions of phage suspensions. The EOP was determined by calculating the ratio of PFU/ml of the tested strain to the reference strain. Some phage strain combinations showed lysis zones when undiluted phages were tested, whereas no plaques were observed with the dilutions. The comparative analysis (Fig. 8) revealed wide host ranges against clinically relevant canine *Pseudomonas* strains. The phages PTLAW1 and PTLAW2 had the broadest host range (66.7% and 68.6%, respectively), followed by JG004, which infected 58.8% of the strains. The phage JG003 was able to infect 31.4% of the *P. aeruginosa* strains. The phage JD05 lysed 27.5% of the tested strains. The isolated temperate phages lysed only 9.8% of the strains. In 5 out of 51 strains (nos. 13, 25, 41, 44, and 57), none of the phages induced a lysis zone.

### Phage propagation, purification and stability

**Fig. 9:**
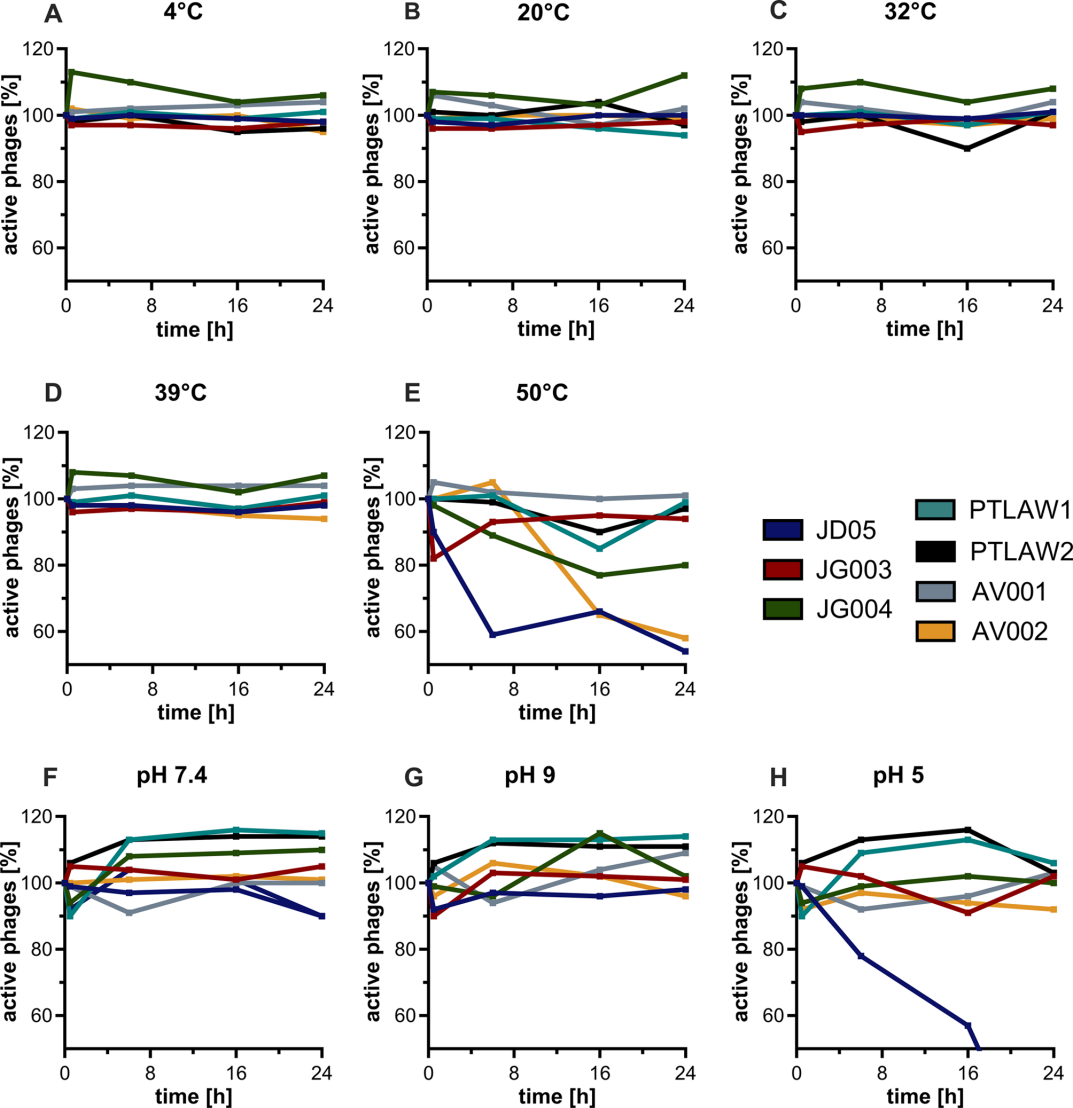
Phage stability at different pH values and temperatures. The temperatures 4 °C (**A**), 20 °C (**B**), 32 °C (**C**), 39 °C (**D**), 50 °C (**E**) and the and the pH values 7.4 (**F**), 9 (**G**) and 5 (**H**) were tested. Shown in % of active phages. The titer was normalized with the titer at time point zero. Phages were incubated for over 24 h under each condition. The titer was testes at timepoints 0, 30 min, 6 h, 16 h, 24 h

The stability of the phages at different pH values (7.4, 9, and 5) and temperatures (4, 20, 32, 39 and 50 °C) was tested over a time period of 24 h (Fig. 9). When the pH values were tested, the phage JD05 was the only phage that showed a loss of activity at pH 5. Three out of 7 phages lost titre at 50 °C.

**Fig. 10:**
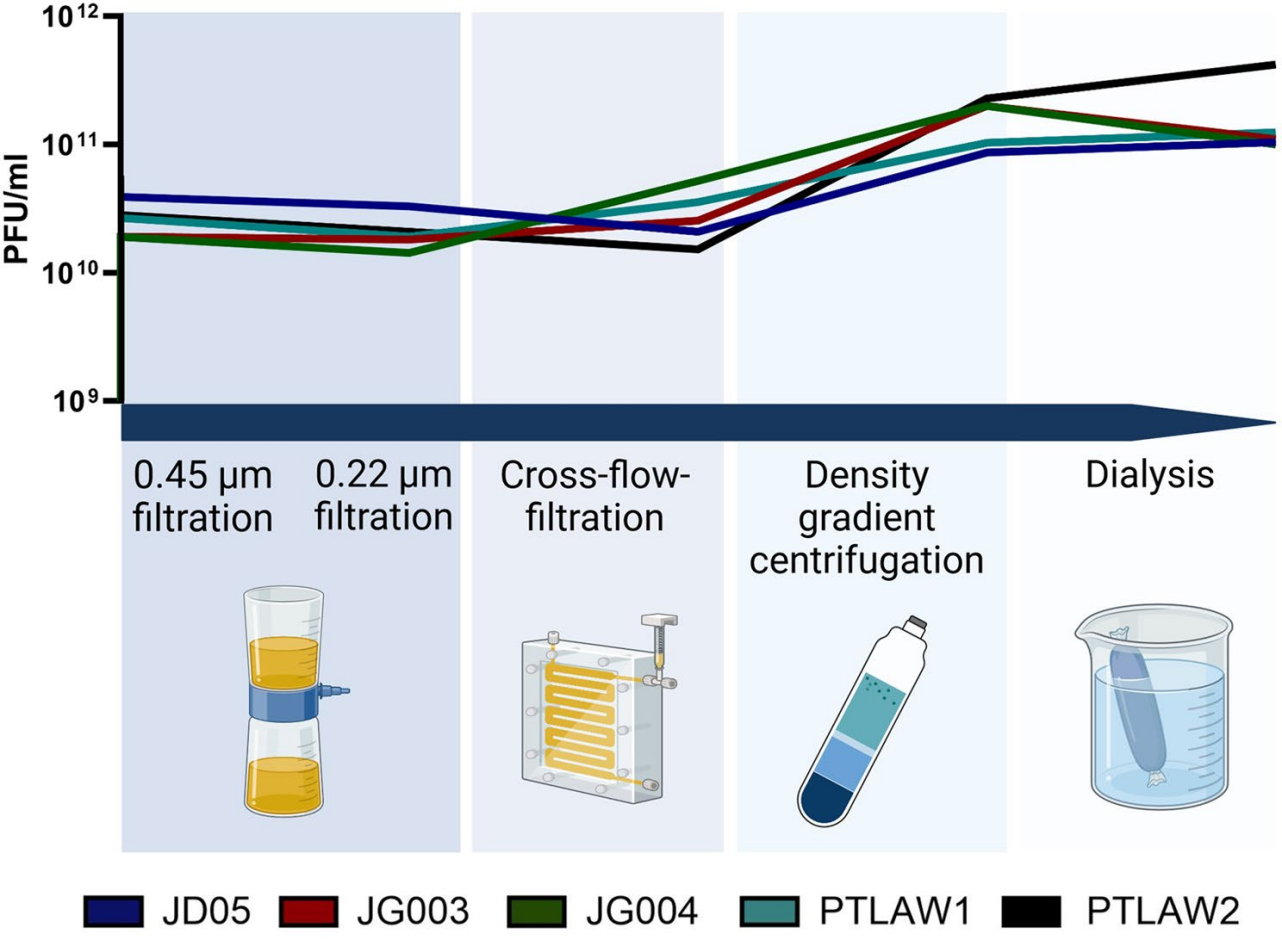
Process stepwise phage titre. Plaque forming unites per ml (y-axis) after dead-end filtration (0.45 μm and 0.22 μm), cross-flow filtration (CFF), density gradient centrifugation (CsCl) and dialysis

The lytic phages suitable for phage therapy were propagated and purified by dead-end filtration, cross-flow filtration, density gradient centrifugation and dialysis. This produced stable high-titre phage preparations of > 10^11^ PFU/ml (Fig. 10). According to their stability in different buffers, the phage suspension was purified in PBS for the phages JG003, and PTLAW1 respectively purified in SM buffer for the phages JD05, PTLAW2 and JG004. All purified phage preparation remained stable over a period of 1.5 years, with no notable titre loss (data not shown) when stored in a glass vial at 4 °C.

### SDS-PAGE and endotoxin-level/phage product safety

**Fig. 11:**
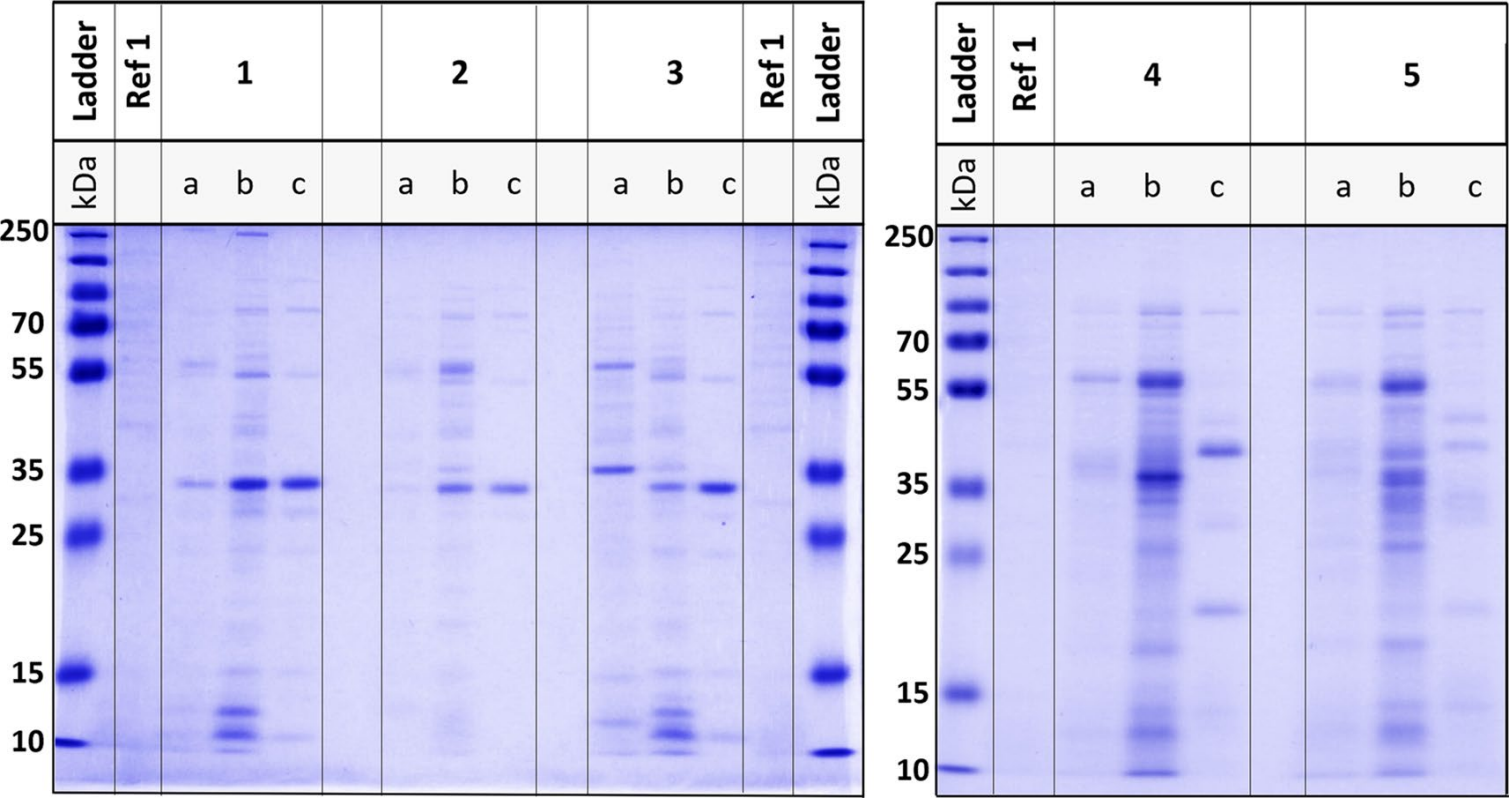
Purification and safety analysis of the final phage preparations. SDS-PAGE analysis of the phage proteins in the steps filtration (**a**), cross-flow (**b**), dialysis (**c**), the bacterial propagation strain (Ref1: IMT45060) and Plus Prestained Protein Ladder (Bio Rad). (1) JD05; (2) JG003; (3) JG004; (4) PTLAW1; (5) PTLAW2

The purification of the phage preparation and the resulting removal of bacterial components containing potential immunogenic components were assessed via SDS‒PAGE and endotoxin measurement (Fig. 11).

On the SDS‒PAGE gel, protein smears over a large range of protein sizes indicate high levels of bacterial protein contaminants. The bacterial propagation strain served as a positive control for bacterial contaminants, while the clear bands in the purified, dialyzed samples presumably represented the viral proteins.

For all phages, a remarkable reduction in smears was observed in the dialyzed sample compared with the sterile filtered or diafiltrated samples (Fig. 11). The original, unmodified SDS-PAGE figures are shown in the Additional file 7. The endotoxin unit (EU) values ranged from 541.67 to 9 044.21 EU/ml. For a dose titre of 10^8^ PFUs, the EU per dose ranges from 0.479 to 8.285 EU. The total treatment dose ranged from 5596.4 to 7541.8 doses, depending on the phage (Table 3).

**Table 3:** Phage product final titre, endotoxin level and estimated number of doses produced for phage therapy

Phage	Titer [PFU/ml]	Endotoxin Units [EU/ml]	Final volume [ml]	Estimated doses with 10^8^ PFUs	EU for a dose of 10^8^ PFUs
JD05	1.05 × 10^11^	541.67	4.5	5596.4	0.523
JG003	1.10 × 10^11^	526.58	5.6	6160	0.479
JG004	1.00 × 10^11^	850.53	6.5	6500	0.851
PTLAW1	1.24 × 10^11^	10243.06	6.1	22,680	8.285
PTLAW2	4.20 × 10^11^	9044.41	5.4	7541.8	2.153

## Discussion

The treatment of *P. aeruginiosa* infections is a big challenge due to the intrinsic resistance, the rise of the acquired antimicrobial resistances and the ability to produce biofilms, which can lead to a treatment failure with commonly used antibiotics. The treatment with phages could be an alternative treatment option. The 7 tested phages showed a broad spectrum of efficacy on the 51 tested canine clinical *P. aeruginosa* isolates. The phages provided by the DSMZ demonstrated the highest host ranges. The phages PTLAW1 and PTLAW2 had the broadest host range (66.7% and 68.6%), followed by the phage JG004 with 58.8% and JG003 with 31.40%. The phage JD05 lysed 27.5% of the strains. These results are rending the phages good candidates for phage therapy. These phages are accessible for further analysis from the DSMZ. The host range is a relevant factor in the efficiency of the phage therapy and is based on a complex interaction between the bacterial receptors and the phage surface receptor. This phage-host specificity is affected by bacterial receptors, the phage surface receptor and the phage recognition mechanism and the genetic compatibility. Monovalent phages, which bind to one receptor, have a narrow host range, while polyvalent phages, that can bind to multiple receptors have a broader host range. Tailed phages are classified in different morphotypes: siphovirus-like, myovirus-like and podovirus-like. For the recognition, the phages use a globular domain at the distal end of the tail fibre, that is composed of different repetitive protein subunits. The number and structure of this subunits differ depending on their morphotype. Phages where these subunits are adaptable and that are using conserved bacterial receptor tend to have a broader host range [59].

Also for optimal cocktail design, it is recommended to combine phages with different receptor specificities, as this reduces probability of cross-resistances between the phages [60]. In our time-killing assay, a bacterial regrowth was observed, suggesting that the bacteria developed resistances to phages. In particular, the combinations of the phages JD05 with JG003 or JG004 showed the same regrowth dynamic to those seen for each phages alone. This may indicate, that, the phages target the same bacterial receptor and a single receptor mutation could lead to a resistance for both. Also the combination of the three phages from different genera, were not able to avoid bacterial regrowth. Additionally, the combination of three phages from different genera was not able to prevent regrowth. Nevertheless, regrowth was observed in all the phage-combinations, indicating a broader resistance mechanism. Supporting this, a study on *P. aeruginosa* showed that cross-resistance is common when phages bind to the same receptor, but mutations in global regulatory genes can also lead to broad resistance by affecting multiple phages [60]. The addition of antibiotics to the phage combination may increase the selective pressure on the bacteria, potentially reducing the phage resistance development, and improving the clinical outcome [61].

Overall, the tested phages encompass *P. aeruginosa* strains commonly found in clinical settings, providing sufficient diversity of phages to enable the use of phage cocktails to combat *P. aeruginosa* infections in patients. The outcome of treatment efficacy in many infections is further dependent on the ability of the phages to degrade the biofilm. The biofilm degradation assay showed that 3 out of 7 phages significantly reduced the biomass of the biofilm with up to 93.38%. These results provide first evidence of the ability of phages to eradicate biofilms. Sequencing analysis of the seven phages, revealed the presence of phage-encoded enzyme genes with potential capacity to degrade biofilms as previously reported in literature, such as glycosidic hydrolases, endonucleases, exonucleases, transglycosylases and pepetidases [62]. These enzymes target and break down components of the extracellular polymeric substances (EPS), disrupting the structural integrity of the biofilm. The hydrolases, an enzyme part of the depolymerases, found in the genome of the phage JG003 can break the glycosidic bond disrupting the biofilm [63]. The exodesoxyribonulease, is known to degrade the extracellular DNA and to destabilize the biofilm. However, such enzymes are also found in the genome of those phages, which could not destroy biofilms in our experiments. The reason for this is likely more complex: the presence of the gene does not guarantee the expression of the genes in our experimental setup and is dependent on environmental signals, host factors and stress signals. Additional experiments on the functionality of the genes for the degradation of the biofilm in our strain would be necessary but are not objective of the current manuscript.

The use of the phages JD05, JG003 and PTLAW1 with antibiotics could improve the therapeutic outcome, as the phages with biofilm-degrading properties can dismantle the extracellular matrix, thereby enhancing the antibiotic’s ability to penetrate deeper into the biofilm layers and effectively target bacteria within [63–65]. Additionally, phages have been shown to disrupt quorum sensing activity, which is crucial for bacterial communication and biofilm formation. This synergistic interaction between phages and specific antibiotics has demonstrated the potential to restore bacterial sensitivity to antibiotics, providing an effective treatment option for infections that are otherwise resistant to antibiotic therapy alone.

For further application of the phages in patients, the phage purification is crucial for enhancing the safety, efficacy, consistency and quality of phage therapy products. Through our purification steps, high-titre phage solutions (> 10^11^ PFU/ml) were produced, which remained stable over a period of 1.5 years. For better stability and reduced phage adsorption, the phage preparations were stored in glass vials [66]. This high level of stability under simple refrigeration conditions (4 °C) for at least 1.5 years highlights the practicality of these preparations for therapeutic use. The number of endotoxin units used for the treatment of 10^8^ PFUs ranges from 0.479 to 8.285 EU. These endotoxin values did not exceed the limit of 5.0 EU/kilogram (kg) in accordance with the FDA’s individual drug products for parenteral use [67]. The phage solutions are produced in a bacterial culture of the host bacterium. The resulting so-called lysate therefore contains a high amount of immunogenic compounds such as endotoxins (i.e., LPS), nucleic acids, flagella, exotoxins and other compounds that must be separated from the phages. These compounds can trigger life-threatening activation of the immune system, such as inflammation and septic shock [68–70], especially if phages are used parenterally. In the future, efforts and initial research achievements are needed to produce personalized therapeutic phages in a cell-free system, which allows genetically well-characterized and cell debris-free phage solutions [71–73]. In recent years, extensive research has been conducted to improve the galenic formulation of phages. However, critical gaps still remain in optimizing formulations for different routes of administration and clinical settings. The stability of the phages in a therapeutical setting can be improved, by incorporating them in gels, creams and hydrogels. Hydrogels, in particular, can provide a moist wound environment that supports phage viability and enables sustained release over time [74].

Monitoring the antimicrobial resistance rates of bacteria originating from companion animal-borne microorganisms is particularly important, especially because of the close relationship between humans and their companion animals [13]. In this study, the prevalence of resistant strains of *P. aeruginosa* from canine samples to different antibiotics was tested. Compared with other studies involving canine otitis samples in other countries [75, 76], different sample types from dogs [77] and veterinary strains from different species [78], the resistance rates reported in our analysis were relatively low. The prevalence of the sample types in our investigations is consistent with the prevalence reported in an evaluation of over 8000 canine samples in Germany from 2019 to 2021 (not published). The same evaluation also revealed similarly low resistance to enrofloxacin, gentamicin and tobramycin. Compared with data for human *P. aeruginosa* isolates published by the German Antimicrobial Resistance Surveillance project (ARS, 2018–2022, inpatients and outpatients) [79] our resistance rates were significantly lower. Because of the high intrinsic resistance to ampicillin, amoxicillin, ampicillin-sulbactam, amoxicillin-clavulanic acid, cefotaxime, ceftriaxone, ertapenem, tetracyclines, trimethoprim, trimethoprim-sulfamethoxazole, chloramphenicol [58, 80–82], the treatment of *P. aeruginosa* remains challenging, despite the low resistance spectrum identified in our analyses. Treatment is further rendered a challenge by the formation of a biofilm.

Although phage therapy can increase the chance of successful and safe treatment, there are also methodological hurdles to overcome. During host range testing with undiluted samples and efficiency of plating (with diluted samples), differences in the lytic zones and the formation of plaques were observed. This phenomenon has been shown in previous studies [83, 84] and highlights the importance of EOP testing to assess the infectivity of phages for therapeutic use since there are different reasons for a non-productive phage lysis: lysis from without, the presence of bacteriocins in the phage lysate, or the presence of prophages harbouring genes coding for phage resistance systems in the genomes of the bacteria [84, 85]. The exclusive testing of high-titre phage preparations could give false positive results on the infectivity of a phage for a bacterial strain, which could be fatal in the case of phage treatment and lead to treatment failure.

For the two phages, isolated from pooled environmental samples, the sequencing analysis revealed that the genomic features of the isolated phages followed a lysogenic path (temperate phage), whereas those of the other phages were significantly related to lysis genes. To the best of our knowledge, the use of temperate phages for phage therapy is not recommended. Temperate phages are pervasive in nature. These mobile genetic elements are usually integrated into the genome of bacteria as prophages and can be transmitted vertically through bacterial cell division. Since phage infections are a constant threat, prophages often encode anti-phage systems, that protect the host cell and themselves from further phage superinfections, described in recent studies also for *P. aeruginosa* prophages [34, 86, 87]. As reported in a recent review [88], these defense mechanisms involve basic mechanisms: blocking phage adsorption or genome entry (Sie system), direct immune defense, which eliminates the phage without harming the bacteria, and abortive infection (abi), where the phage infection cycle is stopped by host cell death. In addition to this defensive role, the use of temperate phages for therapeutic purposes raises further concern. A major issue is the lysogenic conversion, where phage integration leads to a bacterial expression of virulent factors, such as toxins and adhesions proteins, increasing the bacterial pathogenicity (e.g. cholera toxin) [89]. Furthermore temperate phages are involved in the horizontal gene transfer, where phages transfer non-viral genes, including the dissemination of antimicrobial resistance genes, between bacterial populations [90]. Overall, the aspects listed above lead to ethical and safety concerns regarding the use of temperate phages for phage therapy. These aspects are also supported by the recently published “Guideline on quality, safety and efficacy of veterinary medicinal products specifically designed for phage therapy” (EMA/CVMP/NTWP/32862/2022) from the European Medicines Agency, which stipulates the absence of lysogeny in phage products. Despite these reservations, there is increasing interest in using temperate phages as therapeutics with two main engineering approaches [91]: Modifying the lysogenic phages, to become strictly lytic by removing the integration genes [92], or integrating the phages into the bacterial genome to modify the bacterial gene content. The lytic effect of the phages can lead to a harmful release of endotoxins. This could be avoided by the engineering of phages to express proteins, that interfere with essential bacterial metabolic pathways, leading to a bacteriostatic effect. Also the replacement of bacterial genes through temperate phages with CRISPR-Cas has already been described [93]. Additionally, an immunomodulatory effect is described, in which phages influence different processes such as phagocytosis of bacterial invaders and alteration of immune physiology of mammalian host cells [94].

To prevent the development of resistance of bacteria to phages, they should be applied as a mixture of several phages, so-called phage cocktails. The most suitable phages for therapy can be identified combining the results of phage characterization. For therapeutic purposes, the phage cocktail should preferably contain a phage capable of disrupting the biofilm. It is also advisable to combine phages with low similarity, since it can be assumed that phages with high genomic similarity are likely to target identical receptors on bacteria. The promising results of phage therapy in clinical settings highlight its potential, though challenges such as regulatory approval, phage resistance, and possible immune responses remain to be addressed.

## Conclusion

The investigation of phages revealed promising lytic phages with a broad host range and genomic potential for biofilm degradation. Our findings suggest that phage cocktails combining diverse, well-characterized, cleaned-up lytic phages could provide a viable alternative for managing *P. aeruginosa* infections, especially in cases where antibiotic therapy is ineffective.

Further research into optimizing phage preparations and overcoming biofilm barriers will be critical for advancing phage therapy in veterinary and clinical settings.

## Electronic supplementary material

Below is the link to the electronic supplementary material.

Supplementary Material 1

Supplementary Material 2

Supplementary Material 3

Supplementary Material 4

Supplementary Material 5

Supplementary Material 6

Supplementary Material 7

## Data Availability

The sequences of the bacteriophages have been deposited in the GenBank nucleotide sequence database at the National Library of Medicine, National Center for Biotechnology Information (NCBI).The assigned accession numbers for the sequences are as follows: AV001: PQ349086; AV002: PQ349087; JD05: PQ676539; PTLAW1: PQ879542; PTLAW2: PQ798946; JG003: PV105456; JG004: GU988610.

## Abbreviations

P. aeruginosa: Pseudomonas aeruginosa
ANI: Average Nucleotide Identity
EU: Endotoxin Units
WGS: Whole-Genome Sequencing
DSMZ: German Collection of Microorganisms and Cell Cultures GmbH
WHO: World Health Organization
CLSI: Clinical and Laboratory Standards Institute
MIC: Minimal Inhibitory Concentration
DSMZ: German Collection of Microorganisms and Cell Cultures GmbH
MDR: Multidrug-Resistant
LB: Luria–Bertani
AMK: Amikacin
GEN: Gentamicin
TZP: Piperacillin‒Tazobactam
TOB: Tobramycin
CAZ: Ceftazidime
ENR: Enrofloxacin
IMP: Imipenem
MEM: Meropenem
CIP: Ciprofloxacin
MOI: Multiplicity Of Infection
PFU: Plaque-Forming Units
ICVT: International Committee of Taxonomy of Viruses
PBS: Phosphate-Buffered Saline
