# Supplementary material for: Characterization and purification of Pseudomonas aeruginosa phages for the treatment of canine infections
Source: BMC Microbiol. 2025 May 14;25:289. doi: 10.1186/s12866-025-04005-4 (PMC12076904; doi:10.1186/s12866-025-04005-4)
Supplement: Supplementary file 1 — Supplementary Material 1 [file 12866_2025_4005_MOESM1_ESM.pdf]

Additional file 1

Pseudomonas aeruginosa strains used for the phage isolation.

IMT 48843	*P. aeruginosa*
IMT48855	*P. aeruginosa*
IMT49032	*P. aeruginosa*
IMT49041	*P. aeruginosa*
IMT49202	*P. aeruginosa*
IMT49230	*P. aeruginosa*
IMT49214	*P. aeruginosa*
IMT49257	*P. aeruginosa*
IMT24024	*P. aeruginosa*
IMT38122	*P. aeruginosa*
DSMZ25641	*P. aeruginosa*
IMT45060	*P aeruginosa*
