# Supplementary material for: Characterization and purification of Pseudomonas aeruginosa phages for the treatment of canine infections
Source: BMC Microbiol. 2025 May 14;25:289. doi: 10.1186/s12866-025-04005-4 (PMC12076904; doi:10.1186/s12866-025-04005-4)
Supplement: Supplementary file 2 — Supplementary Material 2 [file 12866_2025_4005_MOESM2_ESM.pdf]

Additional file 2: Carbapenemase testing

The strain IMT46516 was tested for carbapenemase production using the mCIM test according to the CLSI standard, resulting in an inhibitory zone of 21 mm.

Multiplex PCR for the genes OXA-48, NDM, KPC, IMP, and VIM was performed, and no expression of these genes was detected.
