# Supplementary material for: Characterization and purification of Pseudomonas aeruginosa phages for the treatment of canine infections
Source: BMC Microbiol. 2025 May 14;25:289. doi: 10.1186/s12866-025-04005-4 (PMC12076904; doi:10.1186/s12866-025-04005-4)
Supplement: Supplementary file 3 — Supplementary Material 3 [file 12866_2025_4005_MOESM3_ESM.pdf]

Additional file 3: Antimicrobial susceptibility testing

Results of the antimicrobial susceptibility testing according to the CLSI standards. The qualitative interpretation was according to the according to the canine-specific breakpoints for amikacin, gentamicin, tobramycin, piperacillin/tazobactam, ceftazidime and cat-specific breakpoints for enrofloxacin (CLSI VET01S ED6:2023). For the antimicrobial agents for which no veterinarian-specific breakpoint is defined, human breakpoints (CLSI M100 ED33:2023) were used. No CLSI breakpoints are available for the antimicrobial agents marbofloxacin.

		Amikacin		Gentamicin		Tobramycin		Piperacillin/ Tazobactam		Ceftazidime		Enrofloxacin		Marbofloxacin		Imipenem		Meropenem		Ciprofloxacin	
Internal number	IMT-number	MIC	Interpretation	MIC	Interpretation	MIC	Interpretation	MIC	Interpretation	MIC	Interpretation	MIC	Interpretation	MIC	Interpretation	MIC	Interpretation	MIC	Interpretation	MIC	Interpretation
Ref. 1	IMT45060	4	S	2	S	<=1	S	<= 4	S	<= 1	S	>= 4	R	>= 4		2	S	<=0.25	S	2	R
Ref. 2	DSMZ25641-1	<=2	S	<=1	S	<=1	S	<= 4	S	<=1	S	0.5	S	>=0.5		2	S	<=0.25	S	<=0.25	S
1	IMT49203	<=2	S	<= 1	S	<=1	S	<=4	S	0.5	S	0.5	S	>=0.5		1	S	<=0.25	S	<=0.06	S
2	IMT49214	<=2	S	<= 1	S	<=4	S	<=4	S	8	S	1	I	>=0.5		1	S	<=0.25	S	0.5	S
3	IMT49257	<=2	S	<= 1	S	<=4	S	>=4	S	2	S	1	I	1		1	S	<=0.25	S	0.5	S
4	IMT51201	4	S	<= 1	S	<= 1	S	<= 4	S	4	S	2	I	1		2	S	<=0.25	S	<=0.25	S
5	IMT51236	<=2	S	<= 1	S	<= 1	S	8	S	4	S	1	I	<=0.5		1	S	<=0.25	S	<=0.25	S
6	IMT51282	<=2	S	4	I	<=1	S	<= 4	S	<= 1	S	0.25	S	<=0.5		2	S	0.5	S	<=0.25	S
7	IMT51313	<=2	S	<= 1	S	<=1	S	<= 4	S	2	S	2	I	<=0.5		1	S	<=0.25	S	<=0.25	S
8	IMT51315	<=2	S	<= 1	S	<=1	S	<= 4	S	4	S	0.25	S	<=0.5		2	S	<=0.25	S	<=0.25	S
10	IMT51339	<=2	S	<= 1	S	<=1	S	8	S	4	S	>= 4	R	>= 4		1	S	<=0.25	S	1	S
11	IMT51355	<=2	S	<= 1	S	<=1	S	<=4	S	<= 1	S	0.25	S	<=0.5		2	S	<=0.25	S	<=0.06	S
13	IMT51405	<=2	S	<= 1	S	<=1	S	<= 4	S	<=1	S	0.5	S	<=0.5		1	S	0.5	S	<=0.25	S

19	IMT51484	16	R	4	I	<=1	S	8	S	4	S	2	I	2		1	S	<=0.25	S	1	S
20	IMT51485	<=2	S	<= 1	S	<=1	S	8	S	4	S	>= 4	R	>= 4		1	S	<=0.25	S	1	S
21	IMT51540	<=2	S	<= 1	S	<=1	S	<= 4	S	<= 1	S	0.5	S	<=0.5		1	S	<=0.25	S	<=0.25	S
22	IMT51561	<=2	S	<= 1	S	<=1	S	8	S	<= 1	S	0.5	S	<=0.5		2	S	<=0.25	S	<=0.25	S
23	IMT51572	<=2	S	<= 1	S	<=1	S	<= 4	S	<=1	S	>= 4	R	>=4		<=0.25	S	0.5	S	2	I
24	IMT51579	<=2	S	<= 1	S	<=1	S	>= 128	R	>= 64	R	1	I	<=0.5		0.5	S	1	S	<=0.25	S
25	IMT51648	<=2	S	2	S	<=1	S	<= 4	S	2	S	1	I	1		1	S	<=0.25	S	<=0.25	S
27	IMT51667	<=2	S	<= 1	S	<=1	S	8	S	4	S	>= 4	R	>= 4		1	S	<=0.25	S	1	S
29	IMT51753	<=2	S	<= 1	S	<=1	S	<= 4	S	2	S	0.5	S	<=0.5		2	S	<=0.25	S	<=0.25	S
30	IMT51785	<=2	S	<= 1	S	<=1	S	<= 4	S	2	S	1	I	<=0.5		1	S	<=0.25	S	<=0.25	S
31	IMT51854	<=2	S	<= 1	S	<=1	S	<= 4	S	<= 1	S	0.5	S	<=0.5		1	S	<=0.25	S	<=0.25	S
32	IMT51856	>= 64	R	<= 1	S	2	S	8	S	4	S	0.5	S	<=0.5		2	S	1	S	<=0.25	S
33	IMT51881	<=2	S	<= 1	S	<=1	S	<= 4	S	<= 1	S	0.25	S	<=0.5		2	S	<=0.25	S	<=0.25	S
34	IMT51912	<=2	S	<= 1	S	<=1	S	16	I	2	S	>= 4	R	>= 4		1	S	1	S	>=4	R
35	IMT51942	4	S	4	I	<=1	S	<= 4	S	<= 1	S	>= 4	R	>=4		2	S	<=0.25	S	1	S
36	IMT51943	<=2	S	<= 1	S	<=1	S	<= 4	S	<= 1	S	2	I	2		2	S	<=0.25	S	0.5	S
38	IMT52055	<=2	S	<= 1	S	<=1	S	16	I	4	S	>= 4	R	2		2	S	1	S	0.5	S
39	IMT52140	8	I	2	S	<=1	S	8	S	4	S	2	I	<=0.5		2	S	<=0.25	S	<=0.25	S
40	IMT52150	<=2	S	<= 1	S	<=1	S	8	S	<= 1	S	0.5	S	<=0.5		1	S	0.5	S	<=0.25	S
41	IMT52239	<=2	S	<= 1	S	<=1	S	<= 4	S	<= 1	S	0.25	S	<=0.5		<=0.25	S	<=0.25	S	<=0.25	S
42	IMT52265	16	R	8	R	<=1	S	<= 4	S	4	S	0.25	S	<=0.5		1	S	<=0.25	S	<=0.25	S
43	IMT45813	<=2	S	<=1	S	<=1	S	<= 4	S	2	S	1	I	<=0.5		<=1	S	<=0.25	S	0.12	S
44	IMT45875	8	S	8	R	<=1	S	8	S	2	S	2	I	2		<=1	S	1	S	0.5	S
45	IMT46403	<=2	S	<=1	S	<=1	S	8	S	2	S	2	I	<=0.5		2	S	<=0.25	S	0.5	S
46	IMT46516	4	S	<=1	S	<=1	S	>= 128	R	8	R	>=4	R	>=4		>16	R	4	I	>=4	R
48	IMT47443	4	S	4	I	<=1	S	<=4	S	2	S	2	I	1		1	S	<=0.25	S	0.5	S
49	IMT47444	<=2	S	4	I	<=1	S	<=4	S	2	S	2	I	1		1	S	<=0.25	S	0.12	S

50	IMT47445	<=2	S	4	I	<=1	S	<=4	S	1	S	2	I	1		1	S	<=0.25	S	0.12	S
51	IMT47452	32	R	>=16	R	4	S	<=4	S	2	S	2	I	1		<=0.25	S	<=0.25	S	0.5	S
52	IMT47616	<=2	S	<=1	S	<=1	S	8	S	2	S	2	I	<=0.5		2	S	<=0.25	S	0.25	S
53	IMT48112	<=2	S	<=1	S	<=1	S	32	R	2	S	2	I	2		0.5	S	1	S	0.5	S
54	IMT48126	<=2	S	<=1	S	<=1	S	8	S	2	S	0.5	S	<=0.5		<=0.25	S	<=0.25	S	0.12	S
55	IMT48127	<=2	S	<=1	S	<=1	S	<=4	S	2	S	2	I	1		1	S	<=0.25	S	0.5	S
56	IMT48287	<=2	S	<=1	S	<=1	S	8	S	2	S	2	I	2		2	S	<=0.25	S	0.25	S
57	IMT48294	<=2	S	<=1	S	<=1	S	16	I	2	S	0.5	S	<=0.5		1	S	1	S	<=0.06	S
58	IMT48719	8	I	4	I	<=1	S	8	S	2	S	2	I	<=0.5		2	S	<=0.25	S	0.25	S
59	IMT48772	<=2	S	<=1	S	<=1	S	<=4	S	2	S	0.5	S	<=0.5		<=0.5	S	<=0.25	S	0.12	S
60	IMT48913	<=2	S	<=1	S	<=1	S	<=4	S	4	S	<=0.12	S	<=0.5		2	S	<=0.25	S	<=0.06	S
