# Supplementary material for: Characterization and purification of Pseudomonas aeruginosa phages for the treatment of canine infections
Source: BMC Microbiol. 2025 May 14;25:289. doi: 10.1186/s12866-025-04005-4 (PMC12076904; doi:10.1186/s12866-025-04005-4)
Supplement: Supplementary file 5 — Supplementary Material 5 [file 12866_2025_4005_MOESM5_ESM.pdf]

Additional file 4: Phylogenetic analysis of the phages with VICTOR and VIRIDIC

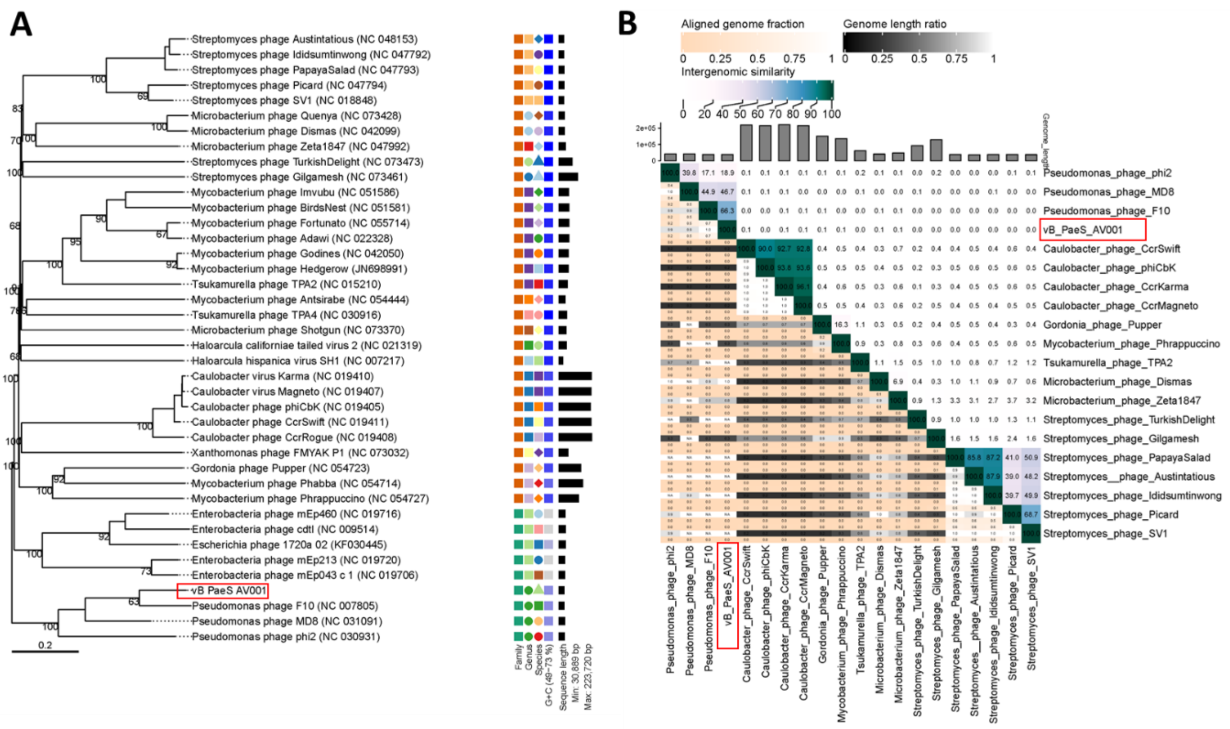

Figure 1: Phylogenetic tree and heat map of AV001 for the closed phage based on BLASTx

(A) Phylogenetic GBDP tree calculated with the formula. The numbers above branches represent GBDP pseudo-bootstrap support values from 100 replications. The branch lengths of the resulting VICTOR trees are scaled in terms of the respective distance formula 0. The color of the symbol indicates the different species, family. (B) VIRIDIC heatmap based on intergenomic similarities between AV001 and closest relatives in BLASTn.

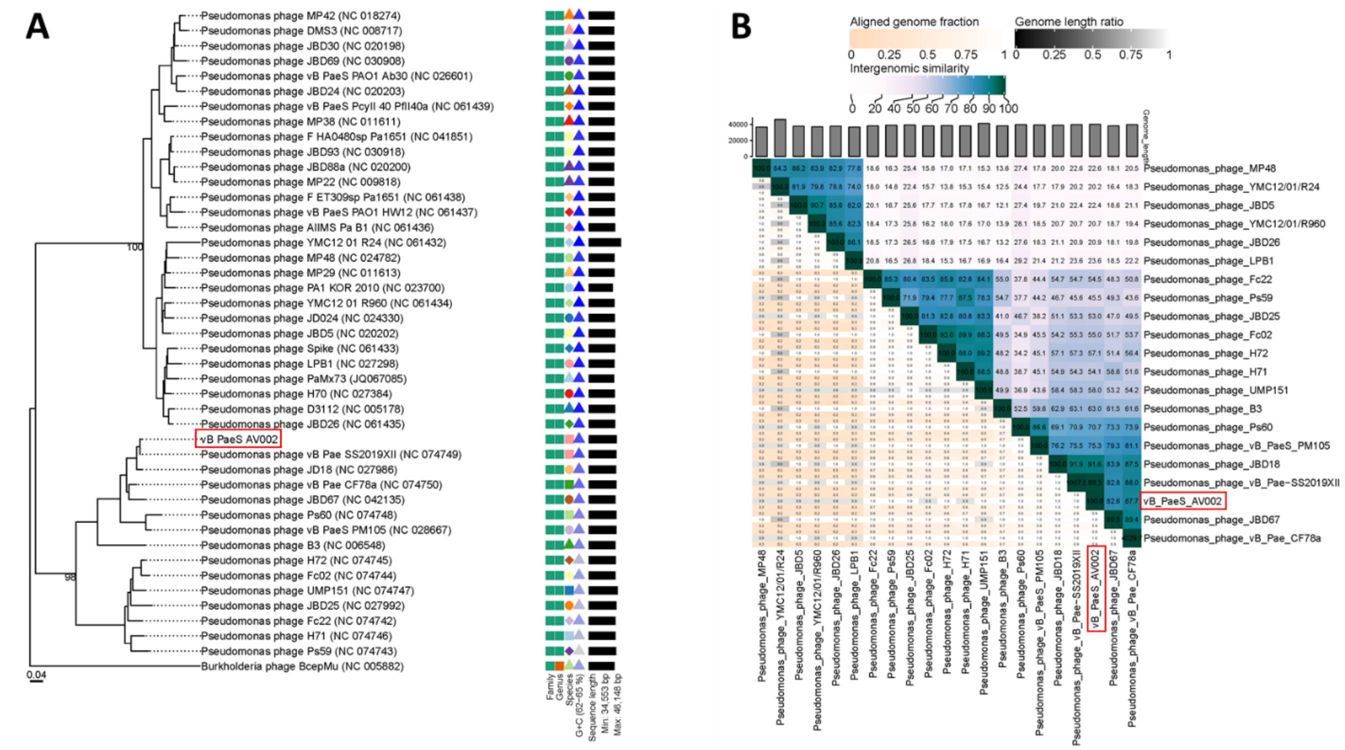

Figure 2: Phylogenetic tree and heat map of AV002 for the closed phage based on BLASTx (A) Phylogenetic GBDP tree calculated with the formula. The numbers above branches represent GBDP pseudo-bootstrap support values from 100 replications. The branch lengths of the resulting VICTOR trees are scaled in terms of the respective distance formula 0. The color of the symbol indicates the different species, family. (B) VIRIDIC heatmap based on intergenomic similarities between AV002 and closest relatives in BLASTn.

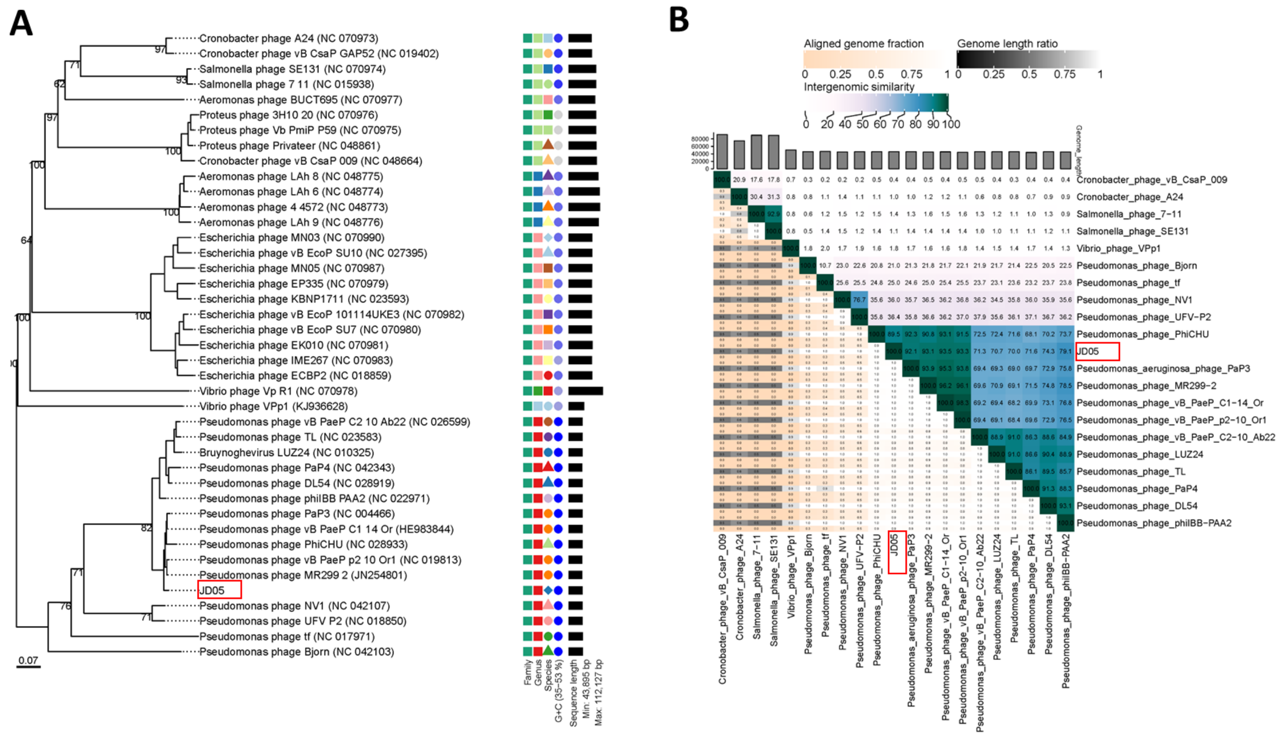

Figure 3: Phylogenetic tree and heat map of JD05 for the closed phage based on BLASTx

(A) Phylogenetic GBDP tree calculated with the formula. The numbers above branches represent GBDP pseudo-bootstrap support values from 100 replications. The branch lengths of the resulting VICTOR trees are scaled in terms of the respective distance formula 0. The color of the symbol indicates the different species, family. (B) VIRIDIC heatmap based on intergenomic similarities between JD05 and closest relatives in BLASTn.

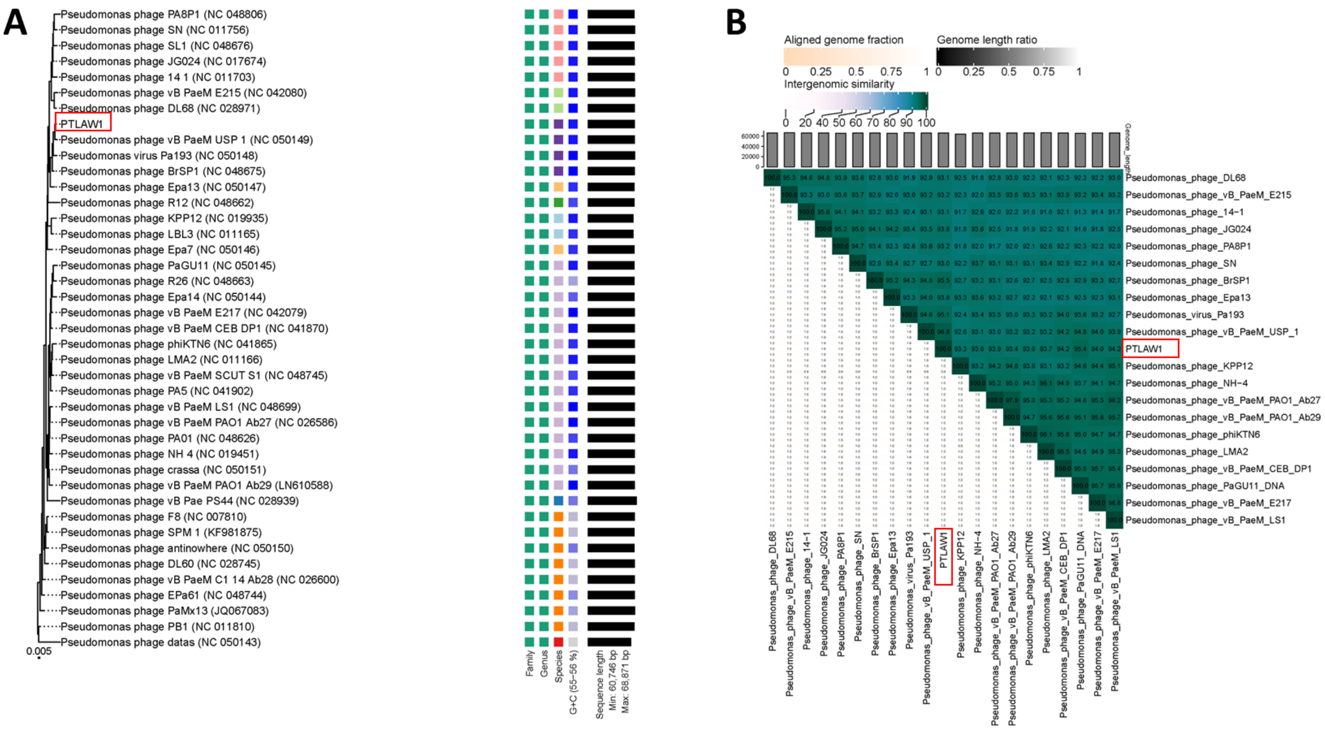

Figure 4: Phylogenetic tree and heat map of PTLAW1 for the closed phage based on BLASTx

(A) Phylogenetic GBDP tree calculated with the formula. The numbers above branches represent GBDP pseudo-bootstrap support values from 100 replications. The branch lengths of the resulting VICTOR trees are scaled in terms of the respective distance formula 0. The color of the symbol indicates the different species, family. (B) VIRIDIC heatmap based on intergenomic similarities between PTLAW1 and closest relatives in BLASTn.

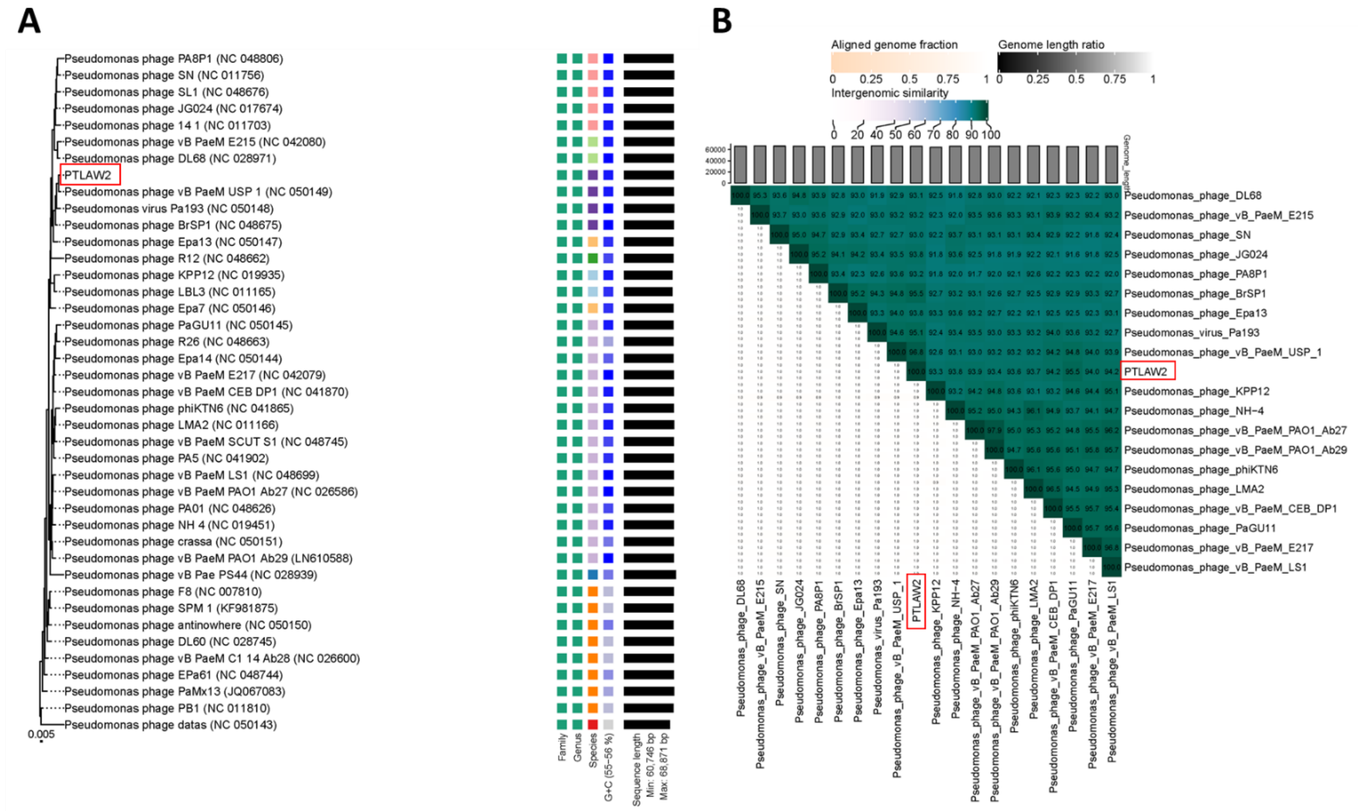

Figure 5: Phylogenetic tree and heat map of PTLAW2 for the closed phage based on BLASTx
(A) Phylogenetic GBDP tree calculated with the formula. The numbers above branches represent GBDP pseudo-bootstrap support values from 100 replications. The branch lengths of the resulting VICTOR trees are scaled in terms of the respective distance formula 0. The color of the symbol indicates the different species, family. (B) VIRIDIC heatmap based on intergenomic similarities between PTLAW2 and closest relatives in BLASTn.

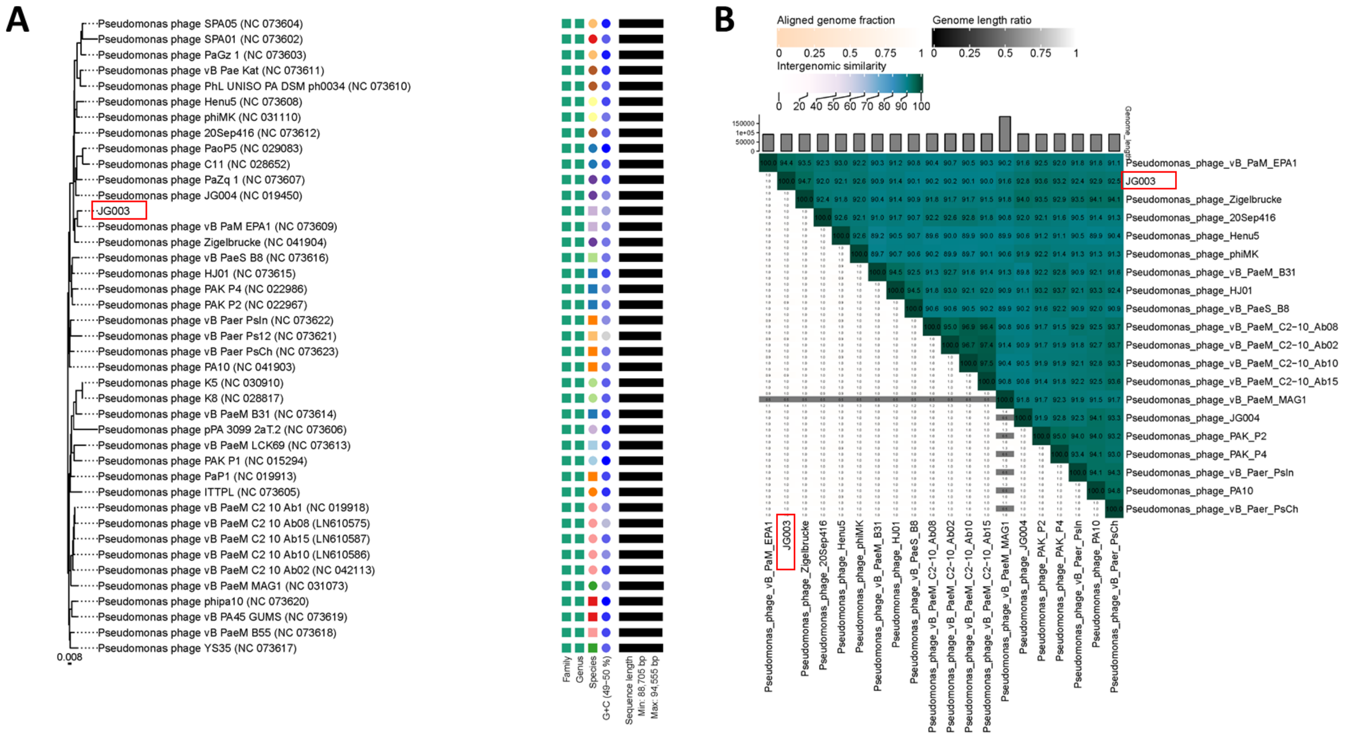

Figure 6: Phylogenetic tree and heat map of JG003 for the closed phage based on BLASTx

(A) Phylogenetic GBDP tree calculated with the formula. The numbers above branches represent GBDP pseudo-bootstrap support values from 100 replications. The branch lengths of the resulting VICTOR trees are scaled in terms of the respective distance formula 0. The color of the symbol indicates the different species, family. (B) VIRIDIC heatmap based on intergenomic similarities between JG003 and closest relatives in BLASTn.

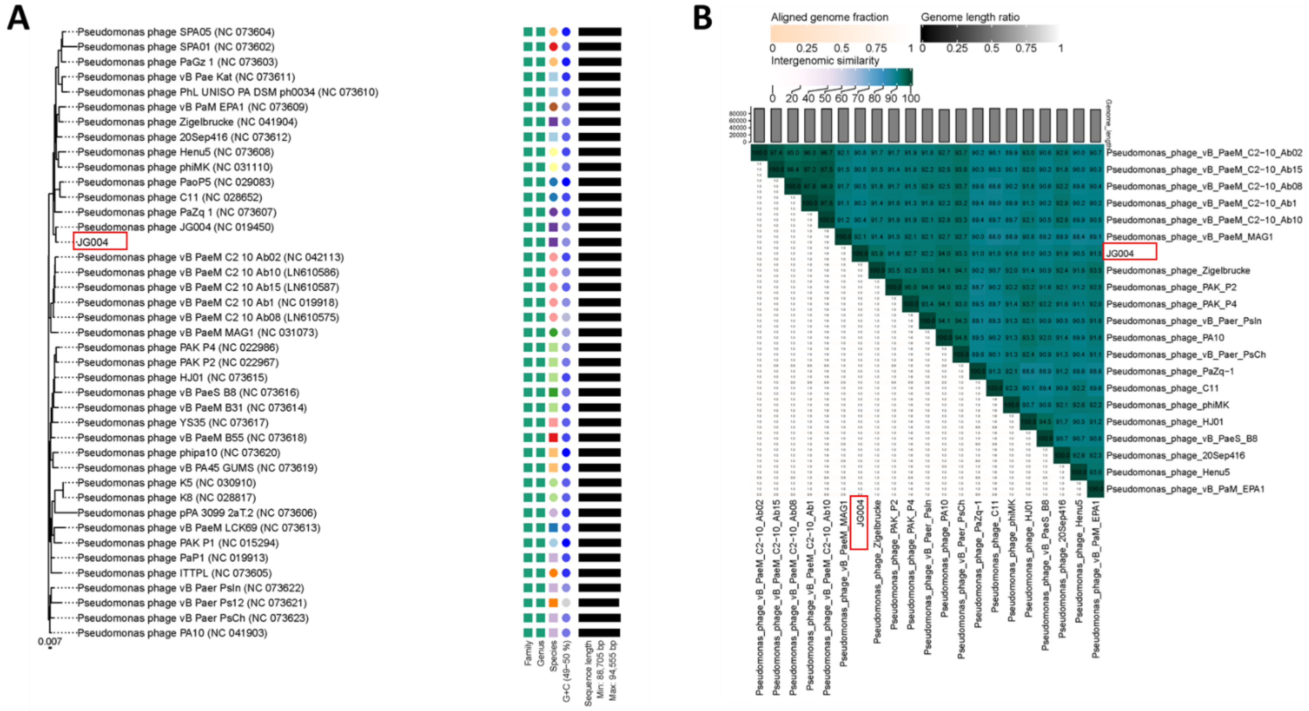

Figure 7: Phylogenetic tree and heat map of JG004 for the closed phage based on BLASTx
(A) Phylogenetic GBDP tree calculated with the formula. The numbers above branches represent GBDP pseudo-bootstrap support values from 100 replications. The branch lengths of the resulting VICTOR trees are scaled in terms of the respective distance formula 0. The color of the symbol indicates the different species, family. (B) VIRIDIC heatmap based on intergenomic similarities between JG004 and closest relatives in BLASTn.
