# Supplementary material for: Characterization and purification of Pseudomonas aeruginosa phages for the treatment of canine infections
Source: BMC Microbiol. 2025 May 14;25:289. doi: 10.1186/s12866-025-04005-4 (PMC12076904; doi:10.1186/s12866-025-04005-4)
Supplement: Supplementary file 6 — Supplementary Material 6 [file 12866_2025_4005_MOESM6_ESM.pdf]

Additional file 6. The kinetics of the time killing curve over 24 hours of the phage combinations

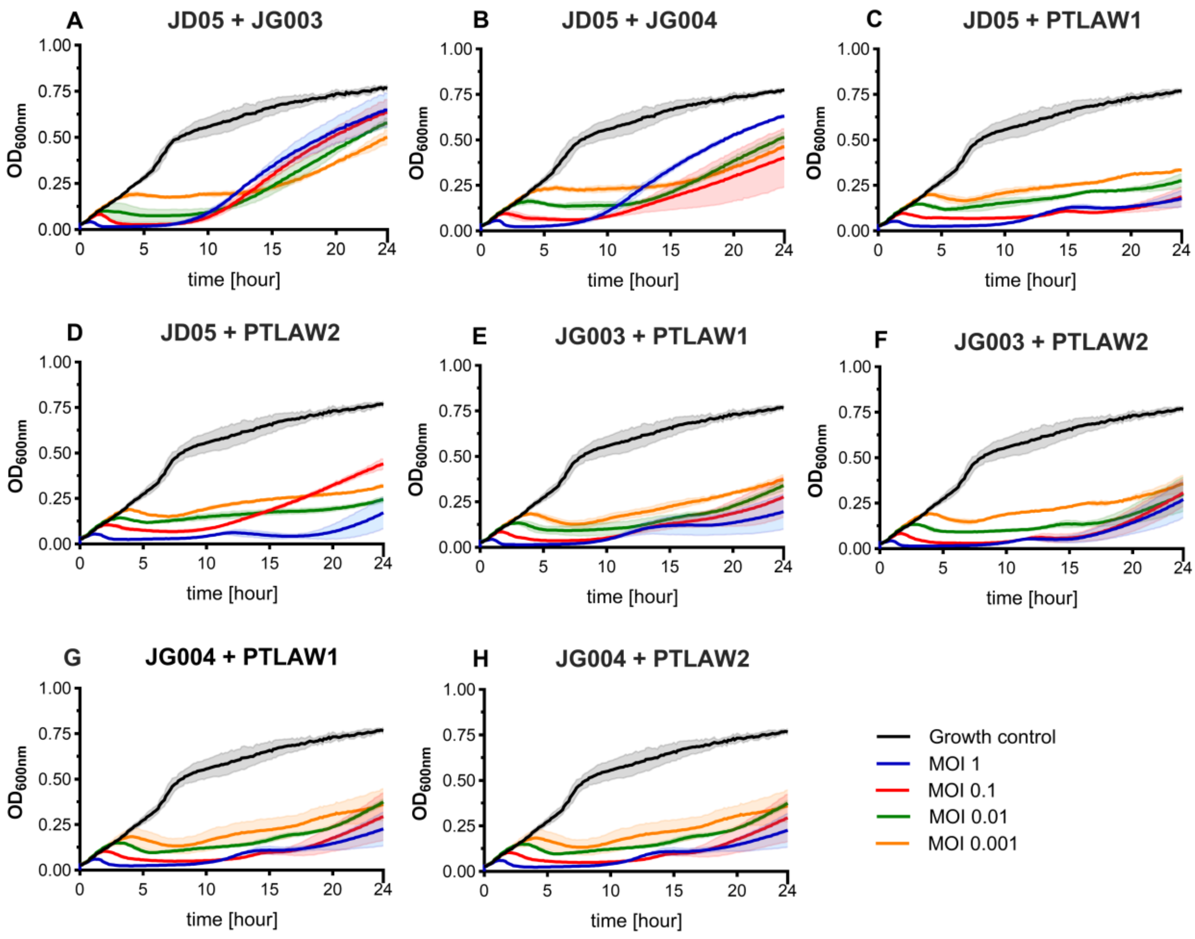

Figure 1: Time killing kinetics of two phage combinations from different genera. The optical density at 600nm was measured every 10 minutes over a 24 hours period. The lytic activity of the phages was tested on their propagation strain with different concentrations, expressed as multiplicity of infection (MOI). (A) JD05 + JG003; (B) JD05 + JG004; (C) JD05 + PTLAW1; (D) JD05 + PTLAW2; (E) JG003 + PTLAW1; (F); JG003 + PTLAW2; (G) JG004 + PTLAW1; (H) JG004 + PTLAW2. Error bars denote the standard deviation of three independent experiments.

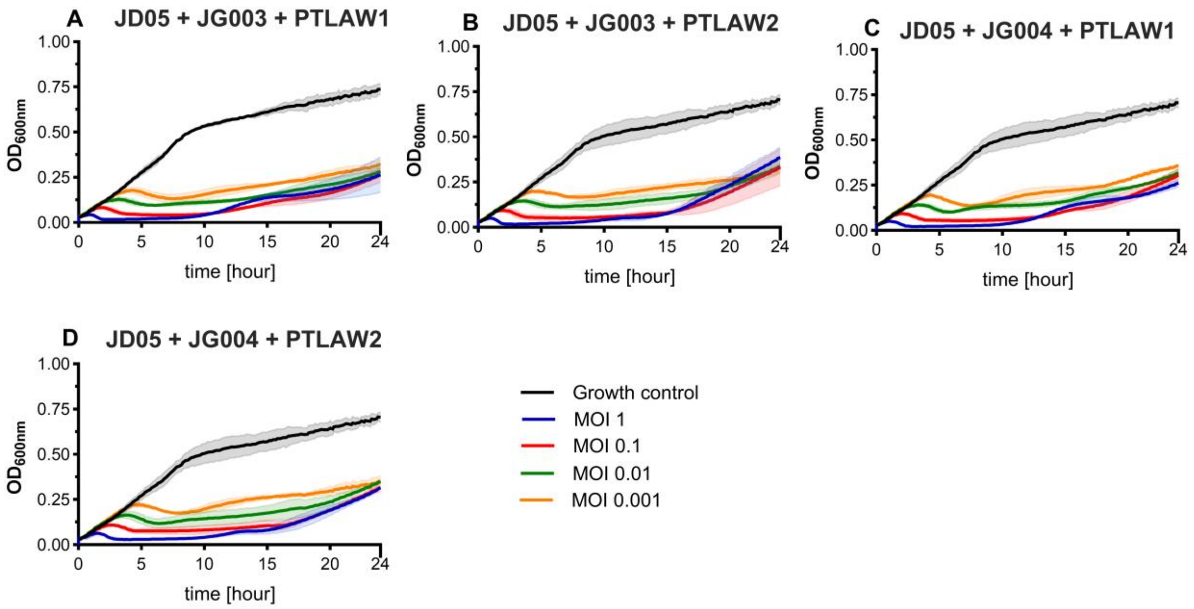

Figure 2: Time killing kinetics of three phage combinations from different genera. The optical density at 600nm was measured every 10 minutes over a 24 hours period. The lytic activity of the phages was tested on their propagation strain with different concentrations, expressed as multiplicity of infection (MOI). (A) JD05 + JG003 + PTLAW1; (B) JD05 + JG003 + PTLAW2; (C) JD05 + JG003 + PTLAW1; (D) JD05 + JG004 + PTLAW2. Error bars denote the standard deviation of three independent experiments.
