# Supplementary material for: Characterization and purification of Pseudomonas aeruginosa phages for the treatment of canine infections
Source: BMC Microbiol. 2025 May 14;25:289. doi: 10.1186/s12866-025-04005-4 (PMC12076904; doi:10.1186/s12866-025-04005-4)
Supplement: Supplementary file 7 — Supplementary Material 7 [file 12866_2025_4005_MOESM7_ESM.pdf]

Additional file 5: SDS-PAGE analysis of the phage proteins in the purification steps

Figures displayed as captured, without modifications.

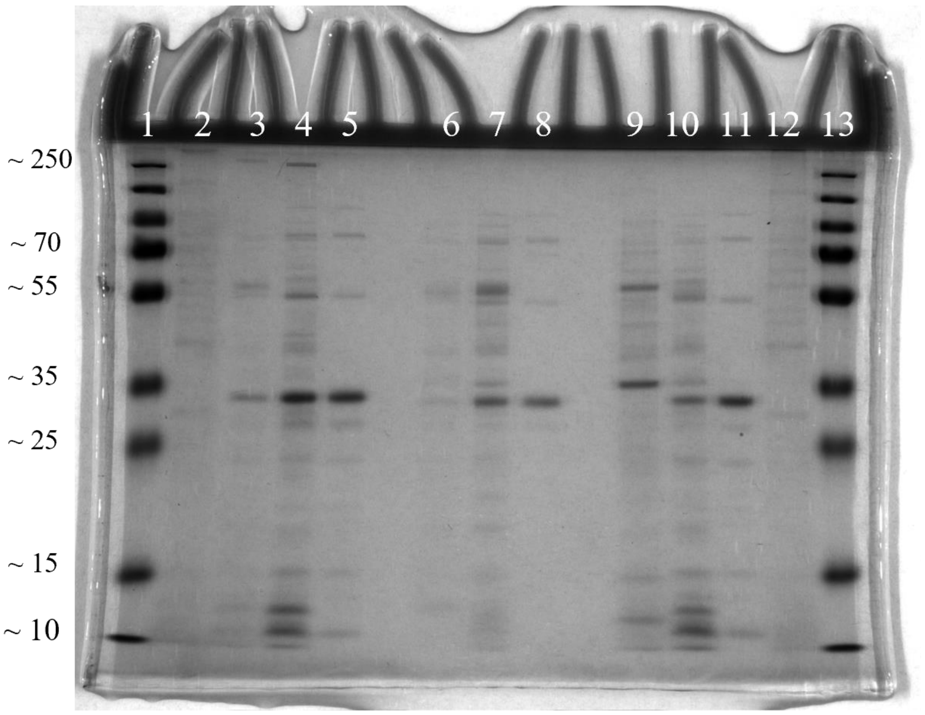

Figure 1: 1D SDS-PAGE analysis of the phage proteins in the purification steps. Figure displayed as captured. 1. Ladder; 2. bacterial Ref1: IMT45060; 3. phage JD05: filtration; 4. phage JD05: cross flow; 5. phage JD05: dialysis; 6. phage JG003: filtration; 7. phage JG003: cross flow; 8. phage JG003: dialysis; 9. phage JG004: filtration; 10. phage JG004: cross flow; 11. phage JG004: dialysis; 12. bacterial Ref1: IMT45060; 13. ladder. The samples were prepared on a 12% polyacrylamide gel stained with PageBlue™ Protein Staining Solution (Thermo Scientific). The Plus Prestained Protein Ladder (Bio-Rad) was used as a molecular weight reference.

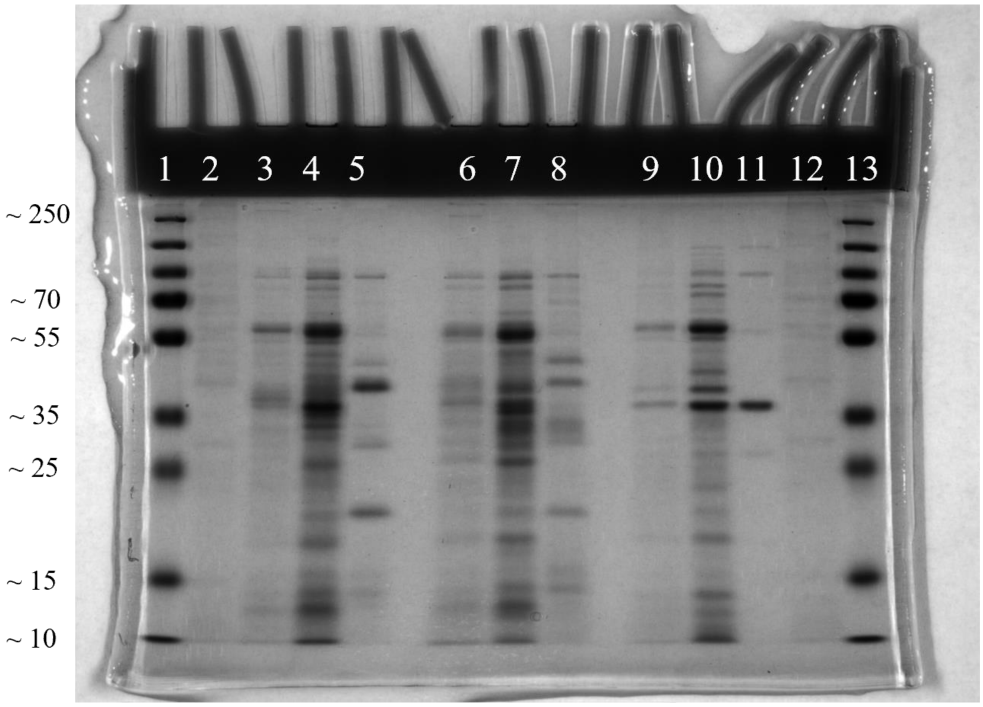

Figure 2: 1D SDS-PAGE analysis of the phage proteins in the purification steps. Figure displayed as captured. 1. ladder; 2. bacterial Ref1: IMT45060; 3. phage PTLAW1: filtration; 4. phage PTLAW1: cross flow; 5. phage PTLAW1: dialysis; 6. phage PTLAW2: filtration; 7. phage PTLAW2: cross flow; 8. phage PTLAW2: dialysis; 9-12. not part of this publication; 13. Ladder. The samples were prepared on a 12% polyacrylamide gel stained with PageBlue™ Protein Staining Solution (Thermo Scientific). The Plus Prestained Protein Ladder (Bio-Rad) was used as a molecular weight reference.
